# Visual adaptation in Lake Victoria cichlid fishes: depth-related variation of color and scotopic opsins in species from sand/mud bottoms

**DOI:** 10.1186/s12862-017-1040-x

**Published:** 2017-08-22

**Authors:** Yohey Terai, Ryutaro Miyagi, Mitsuto Aibara, Shinji Mizoiri, Hiroo Imai, Takashi Okitsu, Akimori Wada, Shiho Takahashi-Kariyazono, Akie Sato, Herbert Tichy, Hillary D. J. Mrosso, Semvua I. Mzighani, Norihiro Okada

**Affiliations:** 1 Department of Evolutionary Studies of Biosystems, SOKENDAI (The Graduate University for Advanced Studies), Shonan Village, Hayama, Kanagawa 240-0193 Japan; 2 Graduate School of Bioscience and Biotechnology, Tokyo Institute of Technology, 4259 Nagatsuta-cho, Midori-ku, Yokohama, 226-8501 Japan; 3 Department of Cellular and Molecular Biology, Primate Research Institute, Kyoto University, Kyoto, Japan; 4 Department of Organic Chemistry for Life Science, Kobe Pharmaceutical University, 4-19-1, Motoyamakita-machi, Higashinada-ku, Kobe, 658-8558 Japan; 5 Department of Anatomy and Cytohistology, School of Dental Medicine, Tsurumi University, 2-1-3 Tsurumi, Tsurumi-ku, Yokohama, 230-8501 Japan; 6 Max-Planck-Institut für Biologie, Abteilung Immungenetik, Corrensstrasse 42, 72076 Tübingen, Germany; 7 Tanzania Fisheries Research Institute (TAFIRI), Mwanza, Tanzania; 8 Present address: Department of Life Sciences, National Cheng Kung University, 701 Tainan, Taiwan; 9 Present address: Foundation for Advancement of International Science (FAIS), Tsukuba, Japan

**Keywords:** Environmental heterogeneity, Color vision, Scotopic vision, Visual adaptation, Cichlid, Lake Victoria

## Abstract

**Background:**

For Lake Victoria cichlid species inhabiting rocky substrates with differing light regimes, it has been proposed that adaptation of the long-wavelength-sensitive (*LWS*) opsin gene triggered speciation by sensory drive through color signal divergence. The extensive and continuous sand/mud substrates are also species-rich, and a correlation between male nuptial coloration and the absorption of LWS pigments has been reported. However, the factors driving genetic and functional diversity of LWS pigments in sand/mud habitats are still unresolved.

**Results:**

To address this issue, nucleotide sequences of eight opsin genes were compared in ten Lake Victoria cichlid species collected from sand/mud bottoms. Among eight opsins, the *LWS* and rod-opsin (*RH1*) alleles were diversified and one particular allele was dominant or fixed in each species. Natural selection has acted on and fixed *LWS* alleles in each species. The functions of *LWS* and *RH1* alleles were measured by absorption of reconstituted A1- and A2-derived visual pigments. The absorption of pigments from *RH1* alleles most common in deep water were largely shifted toward red, whereas those of *LWS* alleles were largely shifted toward blue in both A1 and A2 pigments. In both RH1 and LWS pigments, A2-derived pigments were closer to the dominant light in deep water, suggesting the possibility of the adaptation of A2-derived pigments to depth-dependent light regimes.

**Conclusions:**

The *RH1* and *LWS* sequences may be diversified for adaptation of A2-derived pigments to different light environments in sand/mud substrates. Diversification of the *LWS* alleles may have originally taken place in riverine environments, with a new mutation occurring subsequently in Lake Victoria.

**Electronic supplementary material:**

The online version of this article (doi:10.1186/s12862-017-1040-x) contains supplementary material, which is available to authorized users.

## Background

Lake Victoria harbors more than 500 endemic cichlid species [1, 2]. They are thought to have undergone explosive adaptive radiation during a very short evolutionary period, because Lake Victoria dried up at the end of the Pleistocene and was refilled only 15,000 years ago [3]. Lake Victoria cichlid species share polymorphic nucleotide sites [4–7] due to this short radiation period. Nevertheless, fixed genetic differences were thought to exist between species at loci responsible for the adaptive traits distinguishing the various forms from one another. The long-wavelength-sensitive (*LWS*) opsin gene has been identified as one such gene possessing fixed differences among species, and this has been interpreted as an adaptation to contrasting light regimes [8].

Vertebrate visual pigments consist of a light-absorbing component, the chromophore, and a protein moiety, the opsin [9]. Spectral sensitivity is determined by the chromophore [with 11-*cis* retinal (A1-) or 11-*cis* 3-dehydroretinal (A2-derived retinal)] and by the interaction of the chromophore with the amino acid residues that coat the retinal-binding pocket of the opsin in which the chromophore lies [10]. The replacement of A1- with A2-derived retinal in the pigments shifts the absorption to a longer wavelength [11, 12], and the shift is larger in longer wavelength absorbing opsin pigment [11, 12].

The visual systems of African cichlids have been studied extensively [8, 13–21] because vision is important for food acquisition [22–24] and mate choice [18, 25–27]. African cichlids have eight different opsin genes [8, 13, 14, 19], but only a subset of these is expressed in any individual species [20, 28]. Several Lake Victoria species primarily express the repertoire of four opsin genes [20, 28]: short-wavelength-sensitive opsin gene 2a [SWS2A, λmax of pigments with A1-derived retinal (A1 pigments) = 457 nm; λmax of pigments with A2-derived retinal (A2 pigments) = 472 nm] in single cones; middle-wavelength-sensitive opsin genes (RH2Aβ, λmax of A1 pigments = 523 nm and λmax of A2 pigments = 546 nm or RH2Aα, λmax of A1 pigments = 533 nm; λmax of A2 pigments = 555 nm) and LWS opsin genes (λmax of A1 pigments = 544–559 nm; λmax of A2 pigments = 595–611 nm) in double cones for color vision; and rhodopsin (RH1, λmax of A1 pigments = 503 nm; λmax of A2 pigments = 523 nm) in rods for scotopic vision [17, 18, 29].

The opsins of African cichlids are thought to have adapted to different ambient light conditions varying with depth, water color, and turbidity. The first example described was deep-water adaptation of RH1 pigments in cichlid species from two clear-water lakes, Lake Tanganyika and Lake Malawi. An amino acid change from alanine to serine at position 292 (A292S) shifts the peak wavelength absorbance (λmax) by 11–14 nm toward blue light. This shift from the longer (~500 nm) to the shorter λmax (470–490 nm) is an adaptation from shallow-water to deep-water light regimes [16]. This amino acid change has occurred in parallel in several different lineages [16], and reverse change has also occurred in some of these [30].

In Lake Victoria, *LWS* genes from species inhabiting rocky substrates have been investigated. *Neochromis greenwoodi* is distributed along gradients in turbidity. The populations at opposite ends of one gradient fixed different *LWS* alleles, and these alleles were adaptive to light regimes differing in turbidity [17]. In *Pundamilia* species, *P. pundamilia* and *P. nyererei* are found in shallower and deeper water, respectively. In each species, the *LWS* alleles are adapted to the specific ambient light regimes related to water depth [18]. These studies described correlations between adaptive divergence of *LWS* and male nuptial coloration, suggesting that adaptation of the sensory system to different light regimes drives the divergence of mating signals and leads to reproductive isolation [17, 18].

In contrast to rocky substrates, sand/mud bottoms form a continuous environment. Among six species found on sand or mud, the *LWS* and *RH1* alleles have diversified and one particular allele is dominant or fixed in each species [29]. The functions of the *LWS* alleles are also diversified, whereas those of the *RH1* alleles are identical, as shown by absorption measurements of reconstituted visual pigments [29]. A correlation between male nuptial coloration and the absorption of LWS pigments was also observed among six species [29]. However, because the six species were collected from a single locality with low environmental variability, it is still unclear which factors drive genetic and functional diversity of LWS in sand/mud habitats. To address this issue, we compared ten Lake Victoria cichlid species from sand/mud habitats to identify 1) opsin genes with fixed genetic differences, 2) their functional differences, and 3) the adaptive role of the opsin pigments.

## Results

### *LWS* and *RH1* were dominated by one allele in each species

**Fig. 1:**
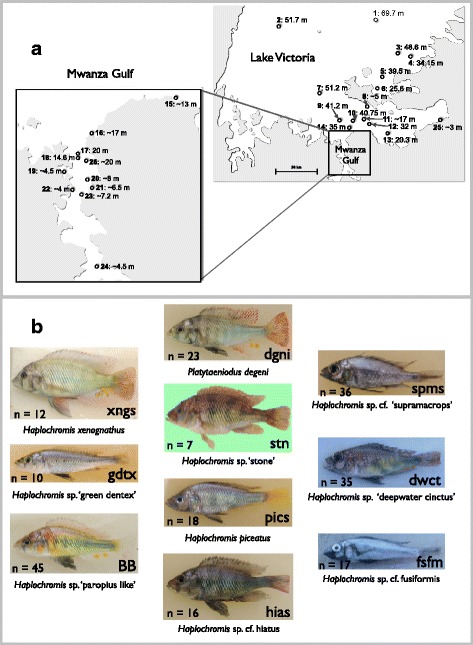
Sampling localities in Lake Victoria and photographs of cichlid species. **a** Map of the southern half of Lake Victoria showing the cichlid sampling localities. Numbers indicate collection localities: 1, Offshore Ukara; 2, Offshore Kerebe; 3, Irugwa; 4, Karaju; 5, Usengere; 6, Kweru; 7, Offshore Lyamwenge; 8, Nansio; 9, Offshore Nyakanyasi; 10, Offshore Makobe; 11, Offshore Mabibi Island; 12, Vessi Island; 13, Offshore Magu Bay; 14, Offshore Makobe; 15, Kayenze; 16, Bwiru Point; 17, Bwiru Point-hippo; 18, Offshore Hippo; 19, Kissenda Bay; 20, South Offshore Chankende; 21, Nyegezi Bay; 22, Nyaruwambu; 23, Nyameruguyu (Python); 24, Offshore Marumbi; 25, Mwabulugu; and 26, Gabalema. **b** Photographs of cichlid species, with abbreviations for species names and the numbers of individuals used in this study for each species. Fish species order is from shallow- (left-top) to deep-water (right-bottom). The maps were drawn by Y. T. based on original source maps: https://www.google.com/maps. Fish photographs were taken by M. I. and S. M

**Fig. 2:**
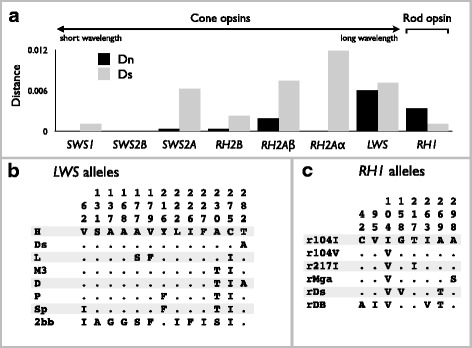
Diversities of opsin genes in Lake Victoria. **a** Genetic diversities of opsin genes. Each column indicates mean distance for synonymous (*gray*) or nonsynonymous (*black*) substitutions for ten species in Lake Victoria. Amino acid alignments of **b** LWS and **c** RH1 alleles. Residue positions are numbered according to the sequences of each opsin gene. The amino acid positions 62, 131, 137, 168, 177, 179, 216, 222, 226, 227, 230, 275, and 282 in LWS correspond to 49, 118, 124, 155, 164, 166, 203, 209, 213, 214, 217, 262, and 269 in bovine RH1. The amino acid positions in cichlid RH1 are identical in bovine RH1. The dots and letters indicate identical and different residues, respectively, compared with the top line

**Fig. 3:**
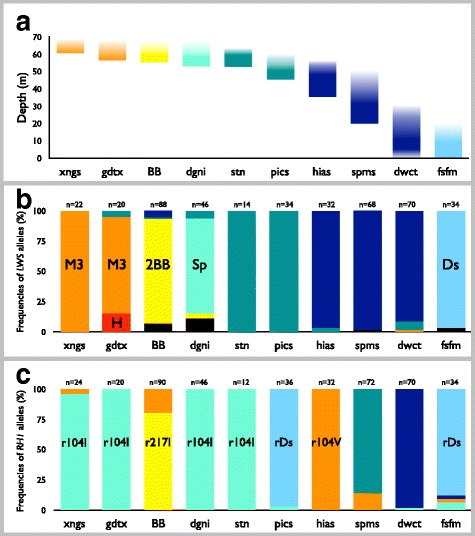
Cichlid species distributions and the frequencies of *LWS* and *RH1* alleles. **a** Species distributions by depth are shown in each column. The positions of the columns of species are identical in (**a**)–(**c**). Frequencies of **b** *LWS* and **c** *RH1* alleles in the ten species. The names of the alleles correspond to those in Fig. 2b and c, respectively. The number of total alleles per species is shown at the top of the columns. Abbreviations for the species names are shown in Fig. 1b

The ten species in this study were distributed from near the surface to the deepest bed (70 m) in Lake Victoria (Fig. 1, Additional file 1: Figure S1a-j). All species were collected from sand/mud substrates. The sequences of seven color opsin genes (*SWS1*, *SWS2A*, *SWS2B*, *RH2Aβ*, *RH2Aα*, *RH2B*, and *LWS*) and one scotopic opsin gene (*RH1*) were determined in two individuals from each of the ten species and used to calculate mean distances of synonymous (Ds) and nonsynonymous (Dn) substitutions (Fig. 2a). High values of Dn were observed in *RH2Aβ*, *LWS*, and *RH1* (Fig. 2a), indicating amino acid variations in *RH2Aβ*, *LWS*, and *RH1*. Of the opsins with high Dn values, species-specific alleles were observed from *LWS* and *RH1*. Therefore, we further determined the sequences of these two genes (Additional file 2: Figure S2 and Additional file 3: Figure S3) from the ten species (in total, 219 individuals) with different depth distributions (Additional file 1: Figure S1a–j). We divided these sequences into eight *LWS* allele and six *RH1* allele groups based on the amino acid sequences (Fig. 2b and c), because the alleles that differed by synonymous substitution were functionally identical. Thus, the sequences that were in the identical allele groups shared the identical amino acid replacements, as shown in Fig. 2b and c. Several allele groups are described in our previous studies [i.e., H, L, M3, and P [17] and Sp, r104V, and r104I [29]]. Based on the allele groups of *LWS* and *RH1*, we compared the depth distribution (Fig. 3a) of allele frequencies of *LWS* (Fig. 3b) and *RH1* (Fig. 3c). The abbreviations for species names are shown in Fig. 1b. In the frequencies of *LWS* and *RH1*, each species was dominated by one allele (Fig. 3b and c). The dominant alleles in *LWS* (M3, P, and D; Fig. 3b) and *RH1* (r104I; Fig. 3c) varied by depth and were shared by species with similar depth distributions. Based on these results, the distributions of *LWS* and *RH1* alleles were likely correlated with depth, and the adaptation of those alleles was inferred.

In addition to Lake Victoria species, we determined the sequences of *LWS* from riverine *Haplochromis* species (*n* = 8).

### Functional diversities of RH1 and LWS pigments

**Fig. 4:**
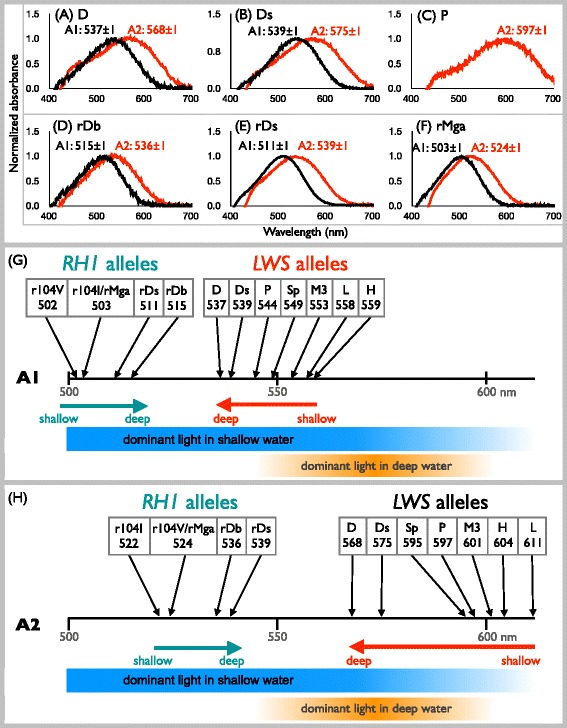
Functional diversities and adaptation of opsin genes in Lake Victoria. **a**–**f** Dark–light difference in spectra of visual pigments. Visual pigments were reconstituted from three *LWS* alleles [(**a**), D; (**b**), Ds; and (**c**), P] and three *RH1* alleles [(**d**), rDb; (**e**), rDs; and (**f**), rMga], with A1- (*black*) and A2-derived (*red*) retinal. The A1 pigments of the P allele were measured previously [18]. **g** Summary of the functional diversities of *LWS* and *RH1* alleles reconstituted by A1- (**g**) and A2-derived (**h**) retinal. The λmax values and allele distributions of L (rocky substratum) [17] and H (sand/mud substratum) [29] were described in previous studies. The distributions of *RH1* and *LWS* alleles are shown by green and orange arrows, respectively. The dominant light wavelength in shallow and deep water [18, 28, 32] is shown by the orange bar

To test whether *LWS* and *RH1* alleles were functionally different, we measured visual pigments that were reconstituted using A1- and A2-derived retinal. The results for several of these measurements were obtained from previous studies [17, 18, 29]. As shown in Fig. 4a–f, we measured newly discovered alleles and an allele (P) that was not measured in the A2 pigments by [18]. We did not obtain the absorption of pigments from 2BB and r217I alleles (Fig. 3b and c) because of the instability of those pigments. The absorption spectra of the pigments were represented by the peak values (λmax), as shown in Fig. 4a–f, with the functional differences of the alleles summarized in Fig. 4g and h. For *RH1* alleles, the λmax values of A1 and A2 pigments ranged from 502 to 515 nm and from 522 to 539 nm, respectively (Fig. 4g and h). The pigments from rDb and rDs alleles were largely shifted toward red (511 and 515 nm for A1 and 536 and 539 nm for A2 pigments, respectively; Fig. 4g and h). For *LWS* alleles, the ranges of λmax values of A1 and A2 pigments varied widely from 537 to 559 nm and from 568 to 611 nm, respectively (Fig. 4g and h). Notably, these wide ranges of λmax values resulted from the replacement of only a small number of amino acids (from H to Sp alleles in Fig. 2b). For example, the A2 pigments from Ds and D alleles were shifted 29 and 36 nm toward blue compared with that of the *H* allele by only one and three replacements, respectively (Figs. 2b and 4h). Hence, the functional diversities of RH1 and LWS pigments were generated by a small number of replacements among alleles.

**Fig. 5:**
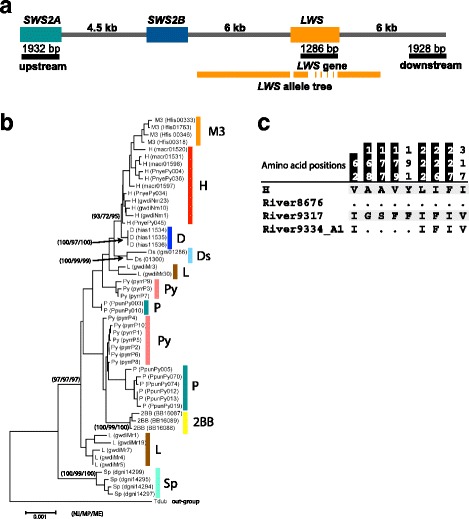
The evolution of *LWS* alleles. **a** The regions used for HKA tests and construction of a *LWS* allele tree are shown by black and orange lines under the genome structure of *LWS* and flanking regions, respectively. **b** Neighbor-joining tree constructed using sequences 5 kbp upstream of *LWS*, *LWS* gene excluding exons, and 3.5 kbp downstream of *LWS*. Bootstrap values are shown for the neighbor-joining tree (left), maximum-parsimony tree (center), and maximum-likelihood tree (right) when the values were 95 or more in any tree. The alleles of *LWS* are shown at the ends of the tree branches. **c** An amino acid alignment of LWS from riverine species. Residue positions are numbered according to the sequences of LWS. The dots and letters indicate identical and different residues, respectively, compared with the top line. The amino acid positions with differences that are found among the alleles from Lake Victoria species are highlighted in black. The *LWS* sequences were determined from four riverine species: *H.* sp. ‘katonga’ (*n* = 2), *H.* sp. ‘katavi’ (*n* = 1), *H.* sp. ‘kitilda-rukwa’ (*n* = 2), and *H.* sp. ‘muzu-rukwa’ (*n* = 3). An alignment of LWS sequences from riverine individuals and sampling localities are shown in Additional file 5: Fig. S4

To reveal the driver for the diversity of *LWS* alleles, we performed HKA tests for heterogeneity between regions (*LWS* gene and up- and downstream regions, shown in Fig. 5a) using the ratio of polymorphism to divergence [31]. Based on the *LWS* allele phylogenetic tree constructed for the *LWS* gene and the flanking regions (5 kb upstream, *LWS* gene without exons, and 3.5 kb downstream, totaling 9492 bp), we used the Sp allele as the out-group for HKA tests (Fig. 5b). The contrast in divergence between the *LWS* gene region (differentiated) and that in the flanking sequences (undifferentiated) was statistically significant (Table 1), with the exception of the D allele because of a small number of polymorphic sites (no site and six sites in up- and downstream regions, respectively). Based on these results, natural selection has acted on the divergence of the *LWS* gene to generate the functional diversity of this gene.

**Table 1:** HKA test for statistical significance (p) of heterogeneity between regions

*LWS* alleles	Up (*SWS2A*)-gene	gene-down	Up (*SWS2A*)-down
2BB	0.0154^*^	0.7876	0.2974
D	0.285	0.2601	0.0995
Ds	0.0016^**^	0.0047^**^	0.6887
M3	0.0200^*^	0.0127^*^	0.8907
P	0.0287^*^	0.0066^**^	0.5814

^*^ *P* < 0.05; ^**^ *P* < 0.01

## Discussion

### Visual adaptation of color and scotopic opsins in Lake Victoria cichlid species

Among eight opsins, amino acid variations and species-specific alleles were observed from *LWS* and *RH1*, indicating no species-specific functional difference in other six opsins. As shown in Fig. 4, we summarized the peak absorption of pigments reconstituted by LWS and RH1 alleles and A1- (Fig. 4g) or A2-derived (Fig. 4h) retinal. The absorption spectra of pigments were measured in this study (RH1: rDb, rDs, rMga; LWS: D, Ds, P-A2 pigment) or previous studies (RH1: r104I, r104V; LWS: H, M3, Sp, P-A1 pigment) [17, 18, 29]. The allele distributions of L (rocky substratum) [17] and H (sand/mud substratum) [29] in shallow water were described in previous studies.

In RH1, the species distributed in deep water (Fig. 3a) possess red-shifted pigments (rDs and rDb) in both A1- (Fig. 4g) and A2-derived (Fig. 4h) retinal. In Lake Victoria, the proportion of long wavelengths in the light spectra increases with depth, with the light in deep water dominated by long wavelengths (i.e., 540–600 nm) [18, 32]. Thus, the absorption shifts toward dominant light in the deep-water RH1 pigments suggest that the functional changes of RH1 pigments from shallow- to deep-water distribution may be an adaptation for efficient absorption of light wavelength (Fig. 4g and h, green arrows). Although the rDs allele predominated in *H. pisceatus*, this species occurs at shallower depths than the other rDs-predominated species, *H.* sp. cf. *fusiformis* (Fig. 3). This difference may be explained by the contrasting distributions of these two species. *Haplochromis pisceatus* and *H.* sp. cf. *fusiformis* specimens were collected from Mwanza Gulf and offshore, respectively (Additional file 3: Figure S1f and j). Water transparency is higher offshore than in Mwanza Gulf [17], and the water turbidity strongly affects the proportion of long wavelengths in the light spectra [17, 28]. Thus, the rDs allele of *H. pisceatus* may be an adaptation to the long-wavelength light that rapidly increases with depth in Mwanza Gulf.

In contrast to RH1 pigments, A1- and A2-derived LWS pigments show different aspects. The λmax values of LWS pigments reconstituted by alleles predominant in species from shallow to deep water (Fig. 3a) gradually shifted toward blue in both A1- (Fig. 4g) and A2-derived (Fig. 4h) retinal. In the case of A1-derived retinal, the λmax values from deep-water species separated from the dominant light in deep water (Fig. 4g, orange arrow), whereas they overlapped with the dominant light in deep water (Fig. 4h, orange arrow) in A2-derived retinal. This suggests that the deep-water A2-derived LWS pigments may adapt to the deep-water light regime, even though the A1/A2 ratio (chromophore usage) of deep water species has not been measured.

In contrast to deep-water, water transmits broad spectra of light (i.e., 400–650 nm) [18, 32] in shallow-water. The λmax values of RH1 pigments reconstituted by alleles predominant in shallow species were located in the middle of wavelength of light, whereas those of LWS A2 pigments were nearly the end of long wavelength light (Fig. 4h). The absorption of LWS A2 pigments may be an adaptation for color discrimination in broad spectra of light in shallow-water.

The A2 chromophore usage was measured [16, 17] and also estimated from MSP data [29] in our previous studies. In our work, the retinal was extracted from the entire eyecup, and the extraction and measurements were performed under conditions in which the unstable A2 retinal could easily degrade. Therefore, the estimation of chromophore usage was more accurate than that determined from measurements of the amount of A2-derived retinal, and we estimated A2 usage from previously described MSP data [20, 33]. The estimated A2 proportion was 20–84% in RH1 and 30–100% in LWS pigments (Additional file 4: Table S1). This suggests that the proportion of A2 was relatively high in Lake Victoria cichlids, and supports the adaptation of A2-derived LWS pigments. The cichlid fish used in the studies of MSP measurements were laboratory-reared fish [20, 33]. In freshwater fish, chromophore usage can change from A1 retinal in bright light to A2 retinal in dark condition [34]. Therefore, our estimation may include the possibility of under estimation of the A2 proportion. Recently, the coexpression of different opsin genes in a single photoreceptor cell was reported [35, 36]. In the case of MSP data for LWS pigments, the coexpression of *LWS* and *RH2Aα* (λmax of A2 pigments = 555 nm) might be able to generate a similar curve to the curve of LWS pigments with A1/A2 mixture. In this case, the MSP data can be explained by LWS and RH2Aα pigments with high proportion of A2 retinal. In RH1, A2-derived retinal usage may also be adaptive, because the λmax values of red-shifted A2-pigments were closer to the dominant light in deep water than for A1-derived pigment. Although the chromophore usage has not been determined, the *RH1* and *LWS* sequences may be diversified for adaptation to different light environments in sand/mud substrates. Measurements of A1/A2 ratio using properly-preserved eye samples to avoid A2 retinal degradation from wild-caught individuals of deep water species will reveal the adaptation of A2-derived opsin pigments to deep water light environments in near future.

The replacement of A1- with A2-derived retinal in the LWS pigments shifted the λmax values to longer wavelength range from 31 nm (D allele) to 53 nm (P and L alleles). Similar to the variation in absorption spectra with the same chromophore (A1- or A2-derived), the variation of λmax value shift by replacement of A1- with A2-derived retinal was also generated by a small number of replacements among alleles. Although we could not find a correlation between amino acid replacements and λmax value shift by replacement of chromophore, mutagenesis, reconstruction, and measurement of visual pigment absorption spectra will reveal the effect of each amino acid replacement in future.

### Evolution of *LWS* alleles in Lake Victoria cichlids

Although Lake Victoria cichlid species show little genetic differentiation [37, 38], the *LWS* sequences were highly diversified (Fig. 2) [8, 17, 18, 29]. The *LWS* sequences from Lake Victoria cichlids were separated into two clades, clade I and clade II, in the phylogenetic tree [8]. However, this tree may not represent the evolutionary process of the *LWS* gene, because the effects of selection cause parallel substitutions in the coding region of this gene. Therefore, we used the up- and downstream flanking regions and intron sequences to construct a phylogenetic tree of *LWS* alleles. We used one clade II allele (2BB) and eight clade I alleles (H, L, M3, P, Py, Sp, D, and Ds) in the analysis. As shown in Fig. 5b, the clade II allele (2BB) formed a clade and diverged from the internal clade of clade I alleles. This suggests that the clade II allele is the derived-type allele. In clade I, the alleles from D and Ds formed each clade, respectively. These alleles possessed alanine at position 282 (282A) that corresponds to position 269 in bovine RH1, and this amino acid caused a large spectral shift toward blue. An alanine at this position has never been found in African cichlids except for these two alleles, raising the possibility that 282A was generated by a new mutation in Lake Victoria. Indeed, the up- and downstream region sequences among three individuals possessing the D allele were identical, suggesting that D is a young allele.

Three deep water species possessed the D allele. This might be explained by either the selection on the D allele in different lineages independently from the ancestral polymorphic alleles, or the selection on the D allele in the common ancestral lineage of three deep water species. The phylogenetic relationship among Lake Victoria cichlids could answer this question. At present, RAD-tag sequencing is the most powerful tool to reconstruct a phylogenetic tree of closely related cichlid species. RAD-tag sequencing was used in a previous study to examine Lake Victoria rock cichlids [7], but only two species in sand/mud substrates were included [39]. Instead of RAD-tag sequencing, we calculated F_ST_ values between each deep water species and the other species using mitochondrial DNA control region sequences among species used in this study. Each of the species pairs between deep water species with D allele and non-deep water species showed the lowest F_ST_ values as follows: *H.* sp. cf. hiatus-*H.* sp. ‘paropius like’ (F_ST_ = 0.01); *H.* sp. cf. ‘supramacrops’-*Haplochromis piceatus* (F_ST_ = 0.05); and *H.* sp. ‘deepwater cinctus’- *H. xenognathus* (F_ST_ = 0.04). This result suggests that three deep water species may not be close relatives, and it was more likely that the selection might have acted on the D allele in different lineages, independently.

By contrast to young alleles, both clade I and II alleles were found outside of Lake Victoria. As shown in Fig. 5c, the allele composition was one clade I allele (River8676) and one clade II allele (River9317), and one recombinant of both sequences (River9334_A1) (Additional file 5: Figure S4C). Among these alleles, seven out of nine amino acid differences were also found among the alleles from Lake Victoria species (Fig. 5c, highlighted in black). These results suggest that the genetic diversity of *LWS* has a riverine origin. Hence, the *LWS* alleles were diversified by using old mutations occurring outside of Lake Victoria and also a new mutation in the lake. The combination of mutations generated by recombination among founder alleles may have been shaped by selection for adaptation to various light regimes in Lake Victoria.

## Conclusions

In this study, we analyzed the nucleotide sequences of eight opsin genes in ten Lake Victoria cichlid species collected from sand/mud bottoms, and showed that the *LWS* and *RH1* alleles were diversified and one particular allele was dominant or fixed in each species. The *LWS* alleles were fixed in each species by natural selection. The absorption of pigments from *RH1* and *LWS* alleles were largely shifted toward different directions, red and blue respectively, in both A1- and A2-derived pigments. In both RH1 and LWS pigments, A2-derived pigments were closer to the dominant light in deep water, suggesting the possibility of the adaptation of A2-derived pigments to depth-dependent light regimes. The usage of A2-derived retinal in freshwater fish has been well known [40], while only a few studies have payed attention and measured the absorption of A2-derived opsin pigments [17, 29]. In this study, we extensively compared the functional differences between A1- and A2-derived pigments in RH1 and LWS, and demonstrated the importance of A2-derived pigments for adaptation to light environments in freshwater fish.

In addition to a correlation between male nuptial coloration and the absorption of LWS pigments in the extensive and continuous sand/mud substrates [29], we revealed that the *LWS* sequences were diversified for adaptation by using old mutations occurring outside of Lake Victoria and also a new mutation in the lake. These visual sensor adaptation and correlation with mating signal (male nuptial coloration) might be a signature of speciation by sensory drive [17, 18], and further studies of the correlation between nuptial colors and visual adaptation will reveal the mechanism of speciation in Lake Victoria cichlid species from sand/mud habitats in the future.

## Methods

### Samples

The Institutional Animal Care and Use Committee of Tokyo Institute of Technology approved the animal protocols and procedures. Ten Lake Victoria cichlid species were examined: *Haplochromis piceatus* Greenwood and Gee 1969, *H. xenognathus* Greenwood 1957, *H.* sp. ‘green dentex’, *H.* sp. cf. *fusiformis* [41–43], *H.* sp. cf. hiatus, *H.* sp. cf. ‘supramacrops’, *H.* sp. ‘deepwater cinctus’, *H.* sp. ‘paropius like’, *H.* sp. ‘stone’, and *Platytaeniodus degeni* Boulenger 1906. The fish were all collected by M. A., S. M., and S. I. M. in 2004–2007. M. A. and S. M. verified all specimen identifications based on Additional file 6: additional text and Additional file 7: Table S2. Information for these species is provided in Additional file 1: Figure S1a–j. Four riverine species were also examined: *H.* sp. ‘katonga’, *H.* sp. ‘katavi’, *H.* sp. ‘kitilda-rukwa’, and *H.* sp. ‘muzu-rukwa’. The localities for each riverine species are shown in Additional file 5: Figure S4.

### DNA sequencing and opsin sequence analyses

Genomic DNA was extracted from caudal fins, pectoral fins, or muscular tissues using a DNeasy Blood & Tissue kit (Qiagen, Hilden, Germany). All tissues were dissected and stored in 100% ethanol until use. Cichlid opsin genes (*SWS1*, *SWS2A*, *SWS2B*, *RH2Aβ*, *RH2Aα*, *RH2B*, *LWS*, and *RH1*) and mitochondrial DNA control region sequences were determined as described previously [15, 17, 29, 44]. The downstream flanking sequences of *LWS* (Fig. 5a) were amplified by two rounds of PCR in a Takara PCR Thermal Cycler Dice Thermal (TakaraBio, Shiga, Japan). The first round amplified 3.5 kbp of DNA fragments of downstream flanking using a pair of primers (LWSdown-longF and LWSdown-longR). The PCR program for the first round of amplification consisted of a denaturation step for 3 min at 94 °C, followed by 30 cycles of denaturation for 1 min at 94 °C, annealing for 1 min at 55 °C, and an extension for 3 min at 72 °C. The second round of PCR used the products of the first PCR as templates, and the DNA fragments were amplified using a pair of primers (LWSB_F26 and LWSdown-longR). The PCR program for the second round of amplification consisted of a denaturation step for 3 min at 94 °C, followed by 25 cycles of denaturation for 1 min at 94 °C, annealing for 1 min at 55 °C, and an extension for 2 min at 72 °C. The PCR products were purified, and their sequences were determined by direct sequencing using an Applied Biosystems Automated 3130 Sequencer. The primers for determination of the sequence of PCR products were LWSB_F27, LWSB_F28, LWSB_R26, LWSB_R31, LWSB_R32, and LWSdown-longR. The positions and the sequences of primers were described by [29]. All sequences obtained were assembled with GENETYX version 10.0.1 or ATGC version 6.0.2 (Genetyx Corporation, Tokyo, Japan).

The sequences of eight opsin genes were determined from two individuals from each of the ten species, with the exception of the sequence for *RH2Aα* from one individual *Haplochromis* sp. ‘paropius like’ (*n* = 1) and the sequence for *SWS2B* from one individual *H.* sp. cf. *fusiformis* (*n* = 1). The mean Ds and Dn values were calculated using MEGA 5.05 [45]. FST values between species were calculated using mitochondrial DNA control region sequences by DnaSP 5.0 [46].

HKA (Hudson, Kreitman and Aguade) tests for heterogeneity were performed using the ratio of polymorphism to divergence [31] between regions: upstream of *LWS* (*SWS2A* gene: 1932 bp) and *LWS* gene (from exon 2 to exon 5, 1286 bp), *LWS* gene and downstream of *LWS* (1928 bp), and upstream (*SWS2A*) and downstream (Fig. 5a). To select the out-group among Lake Victoria *LWS* alleles for HKA tests, a *LWS* locus phylogenetic tree was constructed based on the sequences of the *LWS* gene and the flanking regions (5 kb upstream, *LWS* gene excluding exons, and 3.5 kb downstream, for a total of 9492 bp). The sequences of the *LWS* gene and the flanking regions from three and two individuals with D and Ds alleles, respectively, were determined by the method of [17]. The other sequences were obtained from previous studies [17, 18, 29]. The sequences were divided into alleles and concatenated to include the information of heterozygous sites. The tree was constructed using three methods (Neighbor Joining, Minimum Evolution, and Maximum Parsimony) calculated in MEGA 5.05 [45]. Based on this tree, the Sp allele was selected as the out-group for HKA tests (Fig. 5b).

### Reconstruction and measurement of visual pigment absorption spectra

Production, reconstruction, purification, and measurement of the visual pigments were performed as described previously [17, 47], with minor modifications. Briefly, the sequences of *RH1* (rDs, rDb, rMga, and r217I alleles) were amplified by PCR using genomic DNA of Lake Victoria cichlids as a template with a pair of specific PCR primers [16] designed to produce a fusion protein with a FLAG-tag (Sigma-Aldrich; St. Louis, Missouri, United States) at the C terminus. The LWS-D and -Ds constructs were developed from the LWS-P construct [18] by PCR-based mutagenesis. The amplified DNA fragments were digested with restriction enzymes and cloned into the expression vector pMT5 [48] for LWS or pFLAG-CMV-5a (Sigma-Aldrich; St. Louis, Missouri, United States) for RH1. The visual pigments were reconstituted with A1- and A2-derived retinal, with A2-derived retinal synthesized as described previously [49]. Absorption spectra of the pigment solutions in the presence of hydroxyl-amine (<100 mM) before and after photobleaching were recorded using a spectrophotometer (UV-2400; Shimadzu; Kyoto, Japan), with 5–30 measurements before and after photobleaching. The mean peak spectral values (maximum absorption spectra: λmax) and standard errors were determined from multiple preparations and measurements for each pigment. After reconstitution of the pigments, all procedures were performed under dim red or infrared light (>900 nm) using a digital video camera recorder (DCR-TRV8; Sony) in “night shot” mode or in complete darkness.

### Estimation of A1/A2 ratio

A photoreceptor cell includes both A1 and A2 pigments. MSP measures the absorption spectra of a single photoreceptor cell. The absorption spectra measured by MSP are, therefore, the sum of the absorption spectra of A1 and A2 pigments at a certain A1/A2 ratio. To estimate ratios of A1/A2 pigments in photoreceptor cells, LWS and RH1 standard absorption curves were first constructed using LWS and RH1 absorption curves (Fig. 4a–f) [29]. For each pigment (A1 and A2), the peaks of the curves (λmax) of LWS and RH1 pigments were standardized, with the peak absorbance also standardized. To construct standard curves for A1 and A2 pigments, four absorption curves were averaged and then smoothed. The λmax of standard curves was adjusted to those of *LWS* and *RH1* alleles, with the standard absorption curves then used as template curves (the identical curves with different λmax). For each *LWS* allele, λmax of the mixture of A1 and A2 pigments with a changing A1/A2 ratio was estimated until it adjusted to MSP data. Absorption spectra of LWS pigments measured by MSP in Lake Victoria cichlid species were reported previously [20, 33], and the A1/A2 ratio was estimated for these MSP data.

## Additional files

Additional file 1: **Figure S1.** Short descriptions of the ten species and the frequencies of *LWS* and *RH1* alleles in the populations of (a) *Haplochromis xenognathus*, (b) *H.* sp. ‘green dentex’, (c) *H.* sp. ‘paropius like’, (d) *Platytaeniodus degeni*, (e) *H.* sp. ‘stone’, (f) *H. piceatus*, (g) *H.* sp. cf. hiatus, (h) *H.* sp. ‘supramacrops’, (i) *H.* sp. ‘deepwater cinctus’, and (j) *H.* sp. cf. fusiformis are shown in separate panels. Arabic numerals correspond to those in Fig. 1a, and the depths at each point are described on the right side of the numbers. The size of a pie indicates the number of haplotypes sequenced. The standard sizes of pies are shown at the left bottom. The colored sections of a pie indicate the frequency of the correspondent allele in the standard allele color pie (right bottom). The amino acid differences among allele groups are shown in Fig. 2b (LWS) and Fig. 2c (RH1). The maps were drawn by Y. T. based on original source maps: https://www.google.com/maps. Fish photographs were taken by M. I. and S. M. (PDF 3 kb)

Additional file 2: **Figure S2.** Alignment of all polymorphic sites of *LWS* from the ten species. The nucleotide sites are shown on the top of the alignment. “n” and “s” indicate nonsynonymous and synonymous sites, respectively. Dots indicate the nucleotides that are identical with those in the top line. The allele groups of each sequence are shown on the right side of the sequences. (PDF 64 kb)

Additional file 3: **Figure S3.** An alignment of all polymorphic sites of *RH1* from 10 species used in this study. The nucleotide sites are shown on top of the alignment. “n” and “s” indicate nonsynonymous and synonymous sites, respectively. Dots indicate where nucleotides are identical with those in the top line. The allele groups of each sequence are shown right side of the sequences. (PDF 62 kb)

Additional file 4: **Table S1.** A2 ratio in RH1 and LWS pigments estimated from MSP data. (PDF 119 kb)

Additional file 5: **Figure S4.** Amino acid alignment of LWS from river species. Residue positions are numbered according to the sequences of LWS. The dots and letters indicate identical and different residues, respectively, compared with the top line. The *LWS* sequences were determined from four riverine species: *H.* sp. ‘katonga’ (*n* = 2), *H.* sp. ‘katavi’ (*n* = 1), *H.* sp. ‘kitilda-rukwa’ (*n* = 2), and *H.* sp. ‘muzu-rukwa’ (*n* = 3). (PDF 22 kb)

Additional file 6: Diagnosis and remarks of species used in this study. (DOCX 21 kb)

Additional file 7: General characters of Victoria cichlids used in the study. (XLSX 14 kb)

## Abbreviations

LWS: Long-wavelength-sensitive opsin
RH2: Middle-wavelength-sensitive opsin
SWS: Short-wavelength-sensitive opsin
λmax: The peaks of the absorption curves
