# Supplementary material for: Visual adaptation in Lake Victoria cichlid fishes: depth-related variation of color and scotopic opsins in species from sand/mud bottoms
Source: BMC Evol Biol. 2017 Aug 22;17:200. doi: 10.1186/s12862-017-1040-x (PMC5568302; doi:10.1186/s12862-017-1040-x)
Supplement: Supplementary file 1 — Figure S1. Short descriptions of the ten species and the frequencies of LWS and RH1 alleles in the populations of (a) Haplochromis xenognathus, (b) H. sp. ‘green dentex’, (c) H. sp. ‘paropius like’, (d) Platytaeniodus degeni, (e) H. sp. ‘stone’, (f) H. piceatus, (g) H. sp. cf. hiatus, (h) H. sp. ‘supramacrops’, (i) H. sp. ‘deepwater cinctus’, and (j) H. sp. cf. fusiformis are shown in separate panels. Arabic numerals correspond to those in Fig. 1a, and the depths at each point are described on the right side of the numbers. The size of a pie indicates the number of haplotypes sequenced. The standard sizes of pies are shown at the left bottom. The colored sections of a pie indicate the frequency of the correspondent allele in the standard allele color pie (right bottom). The amino acid differences among allele groups are shown in Fig. 2b (LWS) and Fig. 2c (RH1). The maps were drawn by Y. T. based on original source maps: https://www.google.com/maps. Fish photographs were taken by M. I. and S. M. (PDF 3 kb) [file 12862_2017_1040_MOESM1_ESM.pdf]

Supporting information

(a)

LWS | RH1

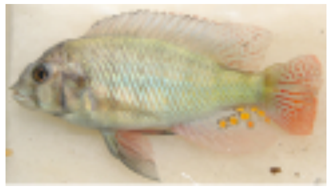

Haplochromis xenognathus Greenwood 1957

This is one of the uniquely shaped haplochromine cichlids in Lake Victoria. The species is easy to identify by the upper jaw protruding over the lower jaw, which is a unique character in haplochromines. The species is widely distributed in the lake, common in Mwanza Gulf in our survey. Habitat is variable, generally rocky shore and border between rocky shore and sandy/muddy floor.

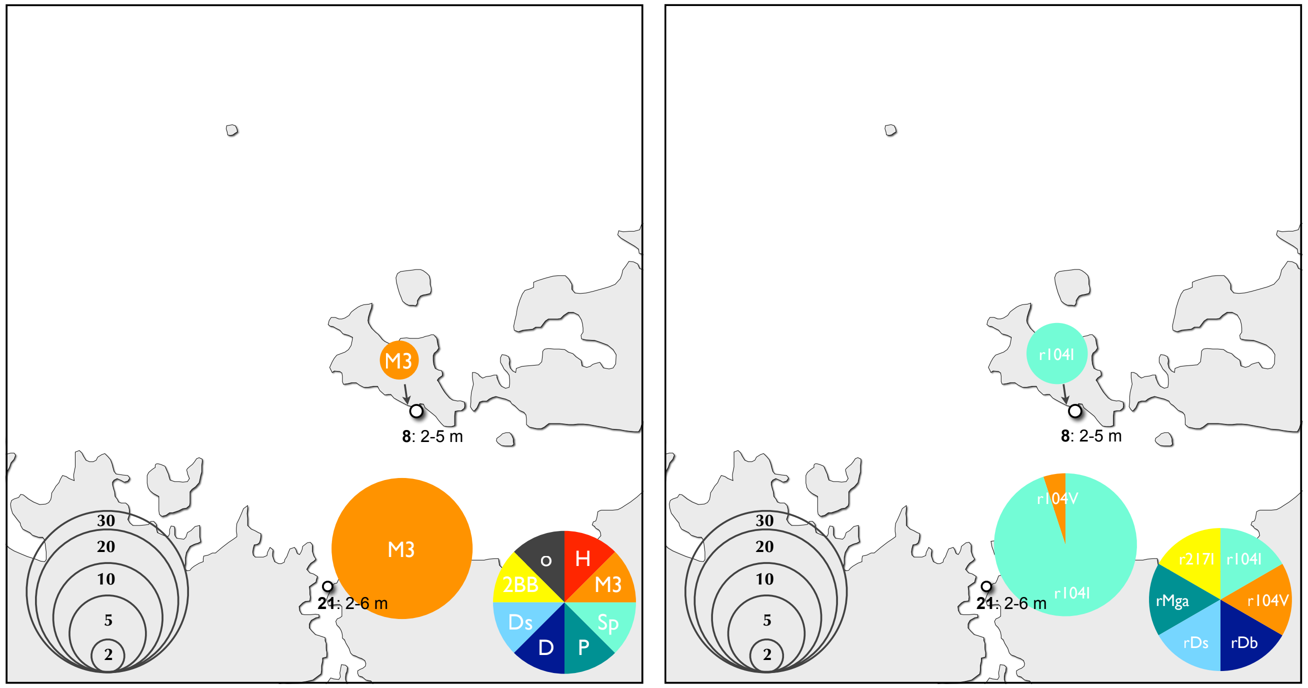

(b)

LWS | RH1

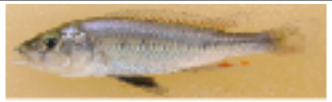

Haplochromis sp. 'green dentex'

This is probably the same species as *Haplochromis* sp. 'green dentex' which was found and named by Haplochromis Ecology Survey Team (HEST) in Leiden University. This is one of few remnant piscivores while most of the piscivores were extinct after Nile perch upsurge. This species is distinguished from the other remnant haplochromines by combination of unicuspid outer teeth in oral jaws (vs. generally bicuspid), extremely slender body, generally smaller body size (see appendix), and pale olive-green coloration with silvery sheen of mature male. This species is widely distributed in southeastern shore of the lake and northern part of Mwanza Gulf, especially abundant in mouth of Mwanza Gulf. Habitat is sandy/muddy floor of sub-littoral zone.

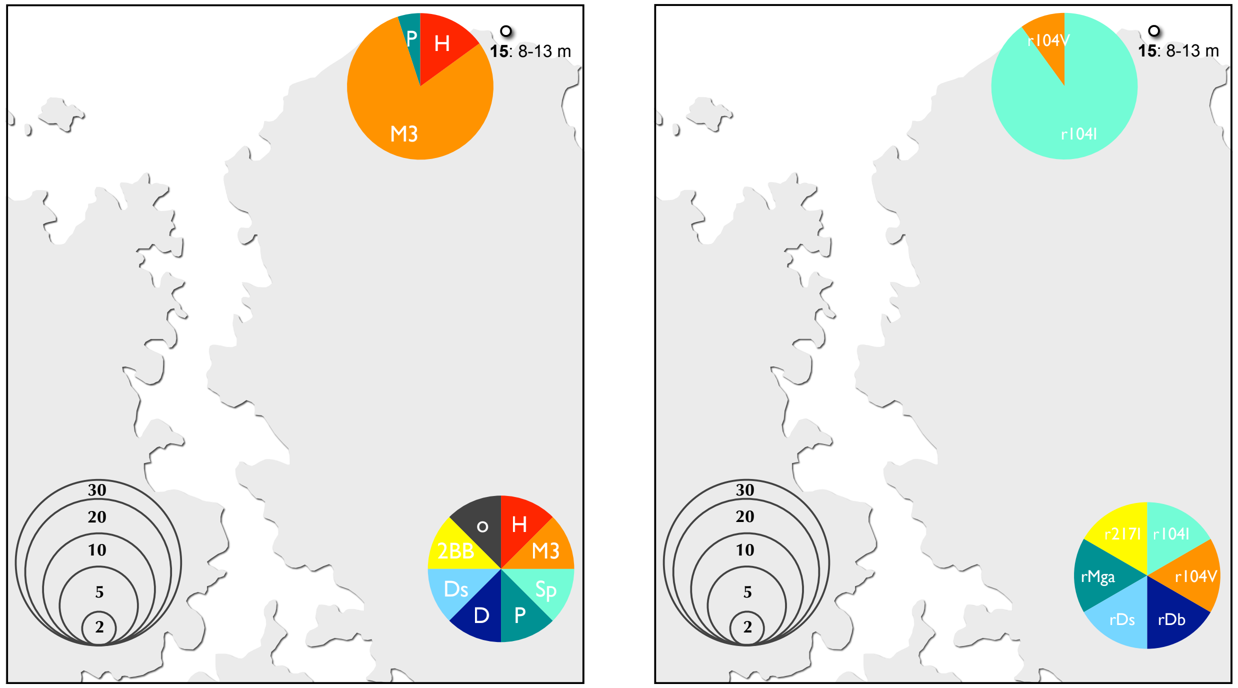

Fig. S1

(c)

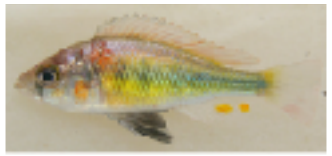

Haplochromis sp.'paropius like'

This is probably the same species as *Haplochromis paropius* Greenwood and Gee, 1969, and *H.* sp. 'broken bar' named by Seehausen et al. (1997) (see Kishe-Machumu et al., 2008) (see appendix). This species is distinguished by small body size, orange body coloration, and broad mid-lateral band in mature male. This species is widely distributed in Lake Victoria, common in northern part of Mwanza Gulf in our survey. Habitat is sandy/muddy floor of sub-littoral zone.

LWS

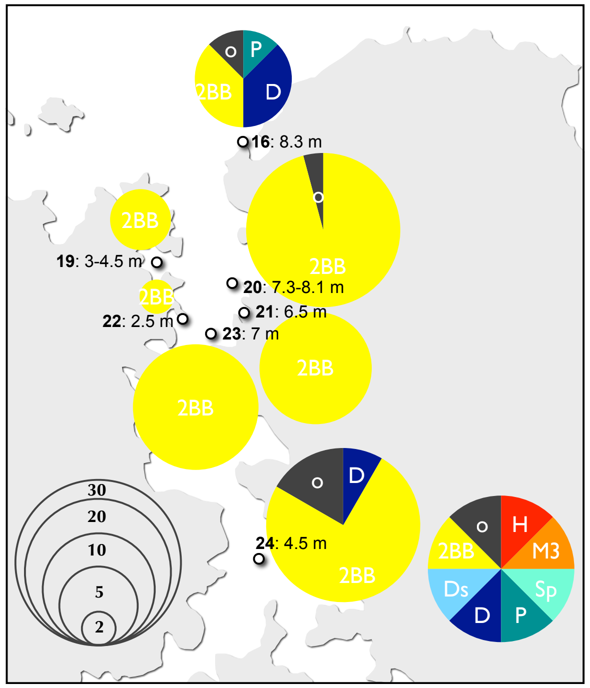

RH1

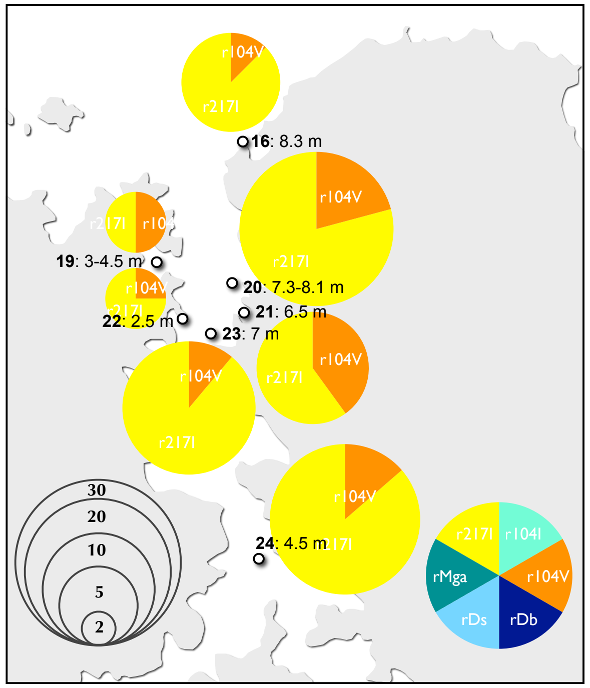

(d)

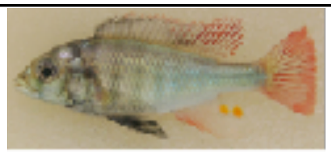

Platytaeniodus degeni Boulenger 1906

This species was reported as extinct in the wild before 1996, but recently is common in the southern part of the lake (Witte et al., 2010). This species is one of the most characteristic haplochromines and easy to distinguish by particularly in the lower jaw. Thus, the posterior end of dental arcade is narrower than medial part, especially in lower jaw, as the result posterior end of lower jaw looks concaved when it is seen from lateral side; shape of teeth band is horseshoe like, whereas U or V shape in the other species. The species is widely distributed in Lake Victoria, common in northern part of Mwanza Gulf in our survey. Habitat is generally littoral sandy/muddy floor or border of sandy floor and cobble stones.

LWS

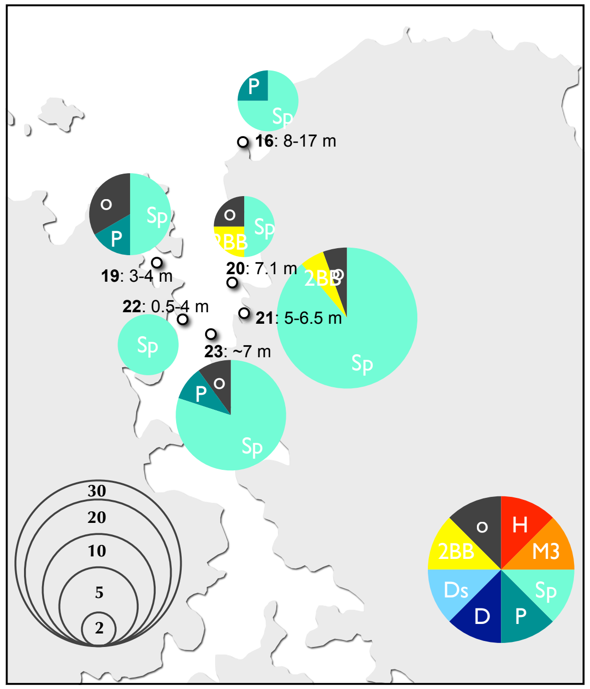

RH1

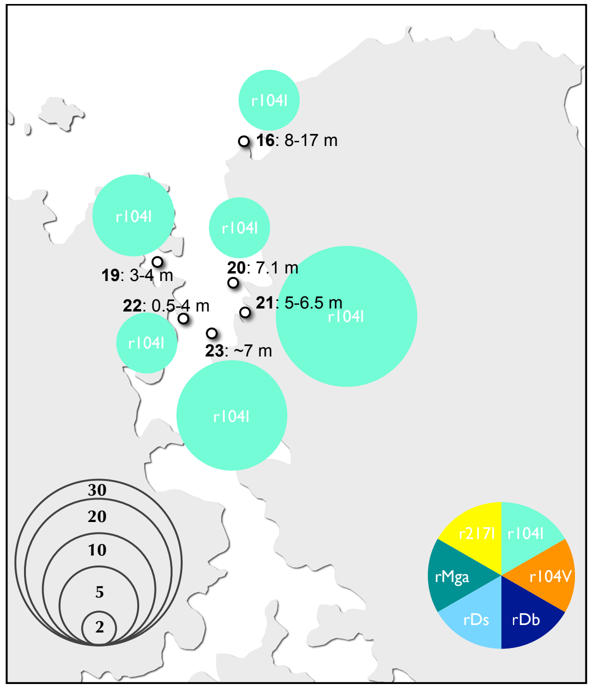

Fig. S1 continued

(e)

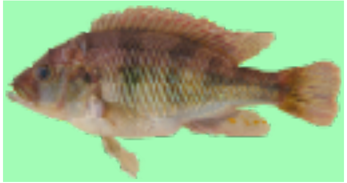

Haplochromis sp. 'stone'

This was named by HEST. This species has hypertrophied lower pharyngeal bone and molariform teeth on the bone, which are specialized to crush shells at the throat. This species is widely distributed and common in northern part of Mwanza Gulf, southwestern coast of Speke Gulf, and several offshore islands in our survey. Habitat is rocky shore in littoral or border of rocky shore and sandy floor in offshore islands. We used individuals from an offshore island population (see right panels).

LWS

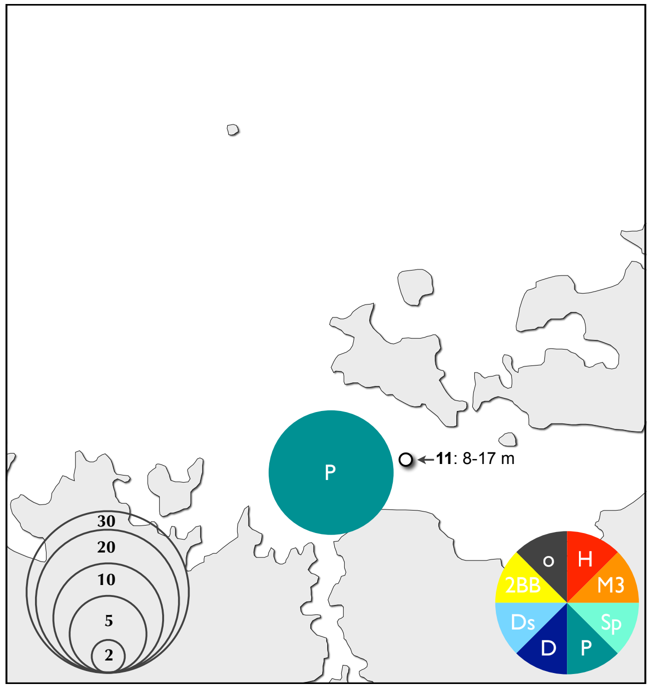

RH1

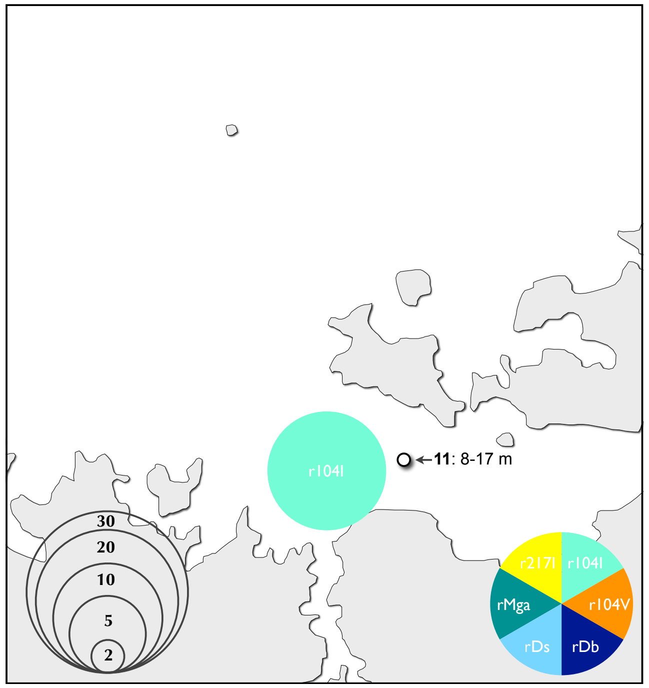

(f)

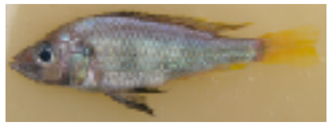

Haplochromis piceatus
Greenwood and Gee 1969

This is one of zooplanktivorous haplochromine inhabiting sandy areas. This species is intermediate between insectivores/ detritivores and zooplanktivores both in body shape and habitat; the former generally inhabit littoral zone and have a moderate body, the latter generally inhabit offshore and have a slender body. This species is distinguished from both of remnant species after Nile perch upsurge by black to dark-green body coloration with yellow to orange unpaired fins (other morphological differences are shown in appendix). Habitat is sub-littoral sandy/muddy floor.

LWS

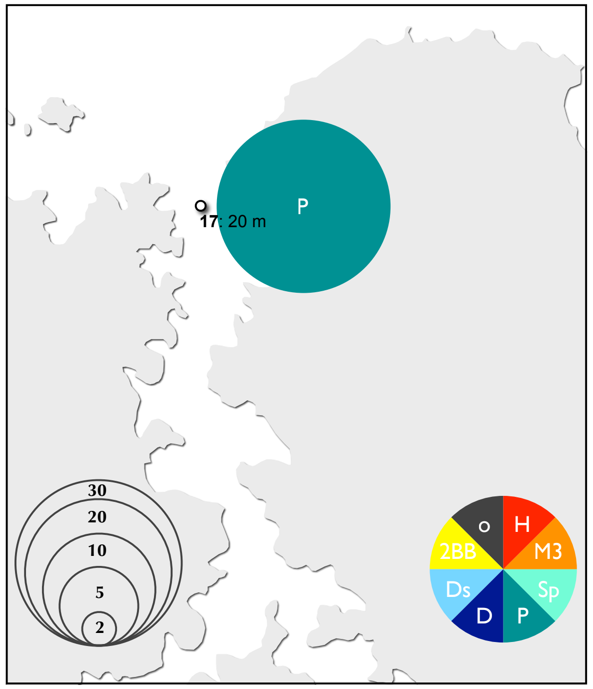

RH1

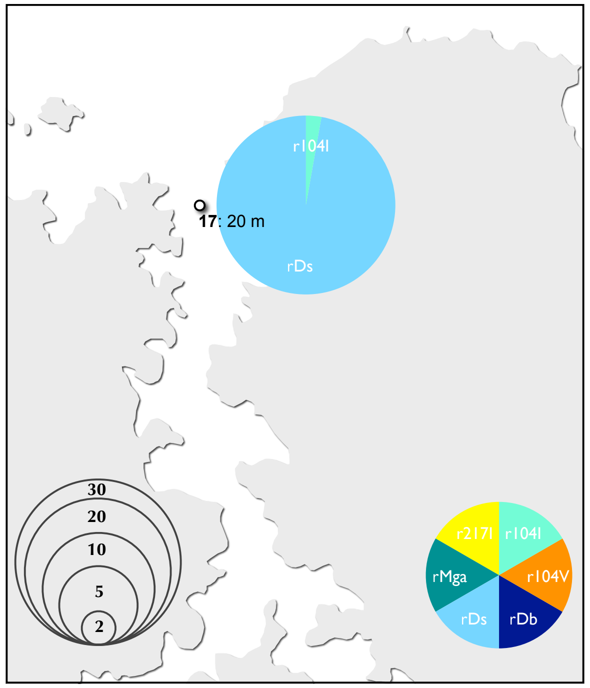

Fig. S1 continued

(g)

LWS

RH1

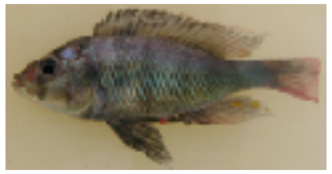

Haplochromis sp. cf. hiatus

This is probably the same species as *Haplochromis hiatus* Hoogerhoud and Witte 1981, but we did not predicate identification at the moment because number of gill filament is slightly less than that from original description, which is diagnosis of the species (see appendix). Additionally, habitat depth was much deeper than that of original description. This species is distributed in mouth of Mwanza Gulf and Speke Gulf, rarely in northern part of Mwanza Gulf. Habitat is offshore sandy/muddy floor.

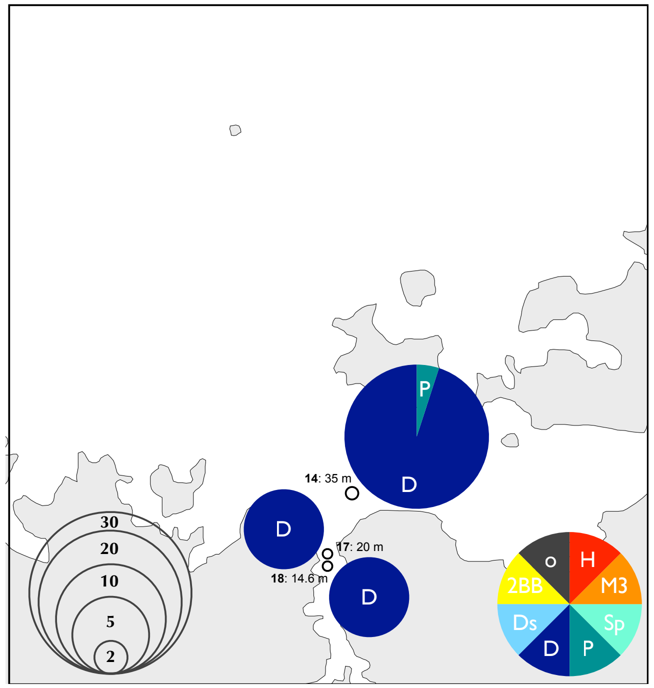

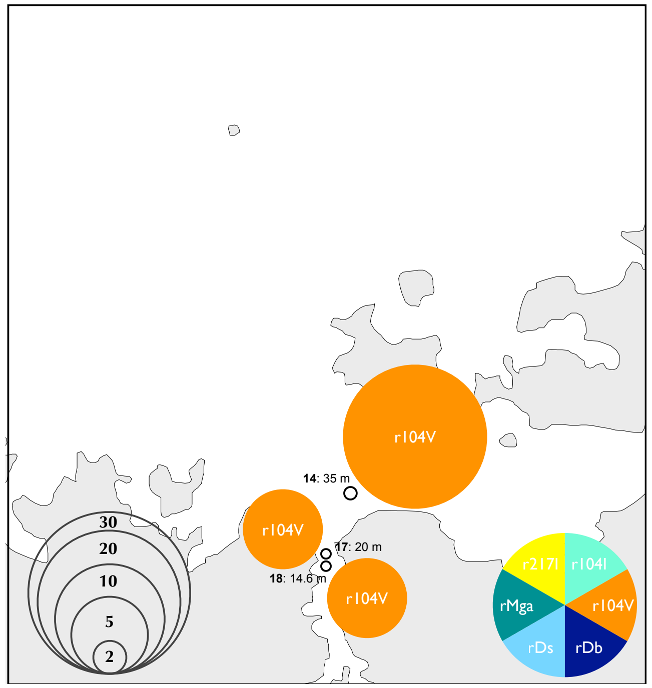

(h)

LWS

RH1

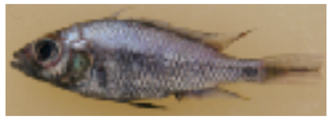

Haplochromis sp. cf. 'supramacrops'

This is probably the same species as *H.* sp. 'supramacrops', which was found by HEST, but the recently collected specimens have relatively small eyes compared with those of past specimens, so we did not predicate identification at the moment (see appendix). This species is widely distributed in Mwanza Gulf, Speke Gulf, and Emin Pasha Gulf in our survey. Habitat is offshore sandy/muddy floor.

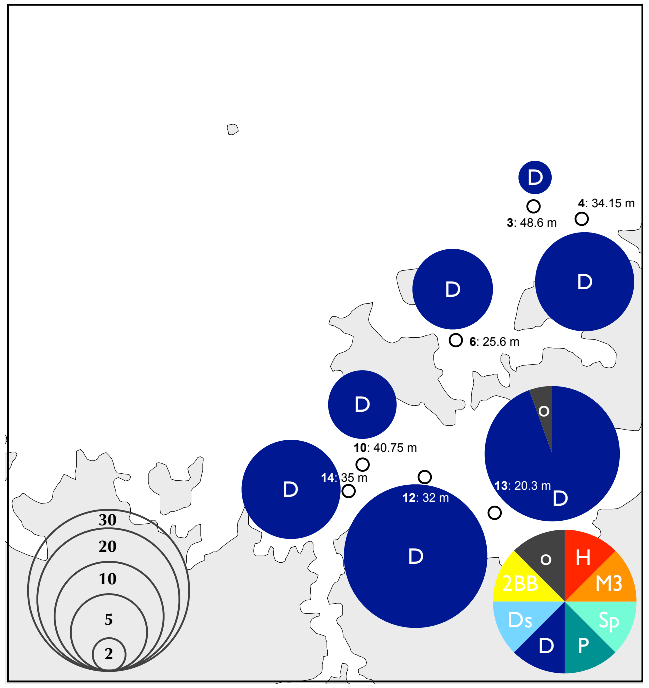

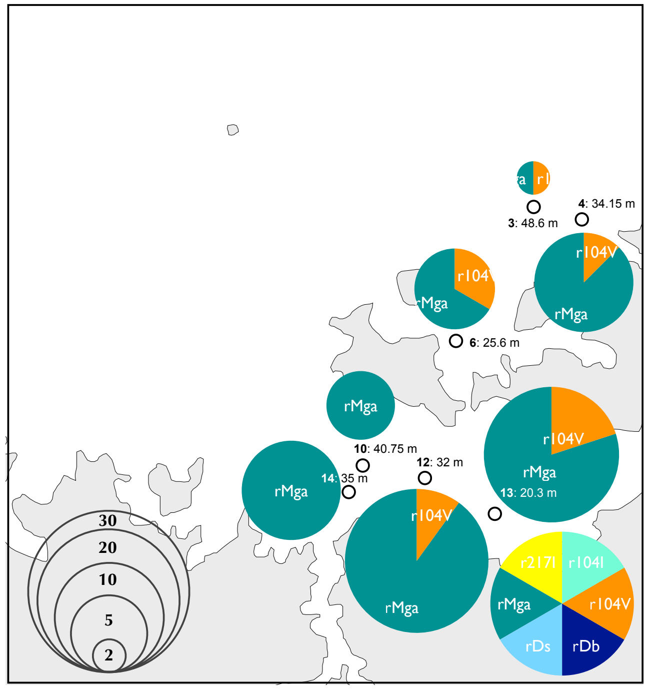

Fig. S1 continued

(i)

LWS

RH1

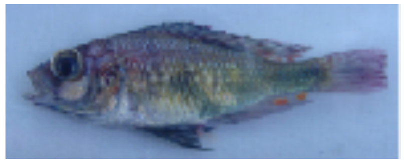

Haplochromis sp. 'deepwater cinctus'

This undescribed species is most likely to insectivores/detritivores, which has not been reported by HEST and other researchers. This species is distinguished from the other remnant haplochromines by small, slender body, weakly bicuspid and/or unequally bicuspid outer teeth in oral jaws, and red body coloration of mature male (see appendix). This species is widely distributed in offshore of southern part of the lake, but distribution in northern part (Kenya and Uganda) is not known. Habitat is offshore sandy/muddy floor.

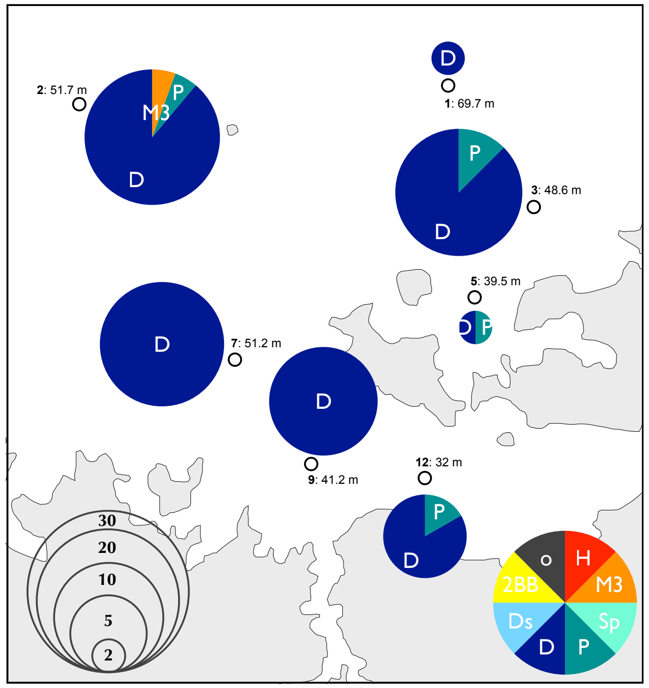

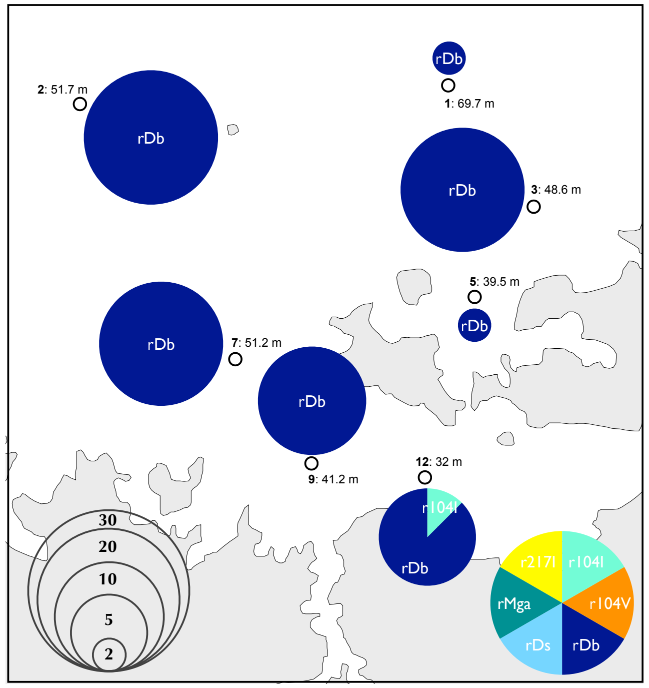

(j)

LWS

RH1

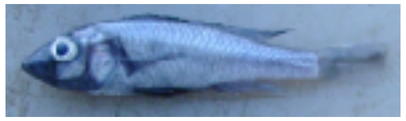

Haplochromis sp. cf. fusiformis

This may be the same species as *H. fusiformis* Greenwood and Gee, 1969, because of extremely slender body, slender and unequal bicuspid outer teeth in oral jaws, and offshore habitat; however we did not predicate identification at the moment, because coloration is different (see appendix). Additionally, our specimens were collected from quite deeper areas (see right panels) in the southern part of the lake, whereas *H. fusiformis* has been collected from moderate depths in northern areas (27-33 m according to original description). Habitat is offshore deep sandy/muddy floor.

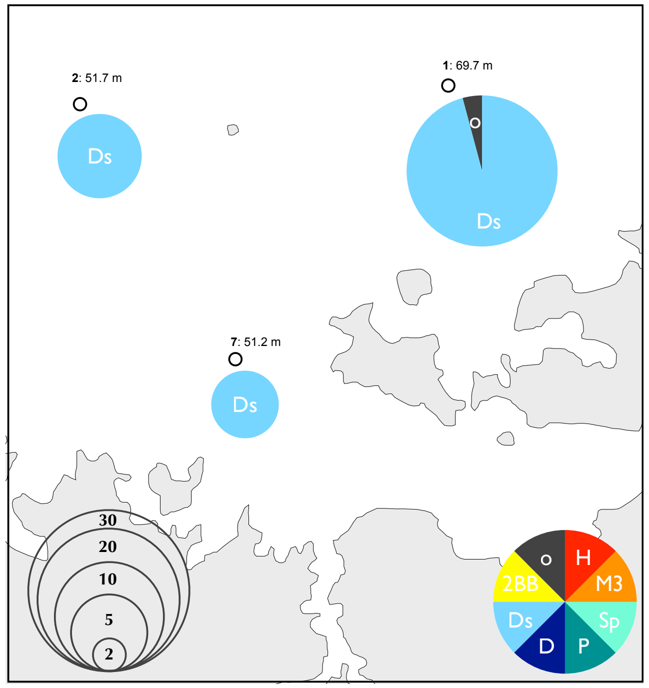

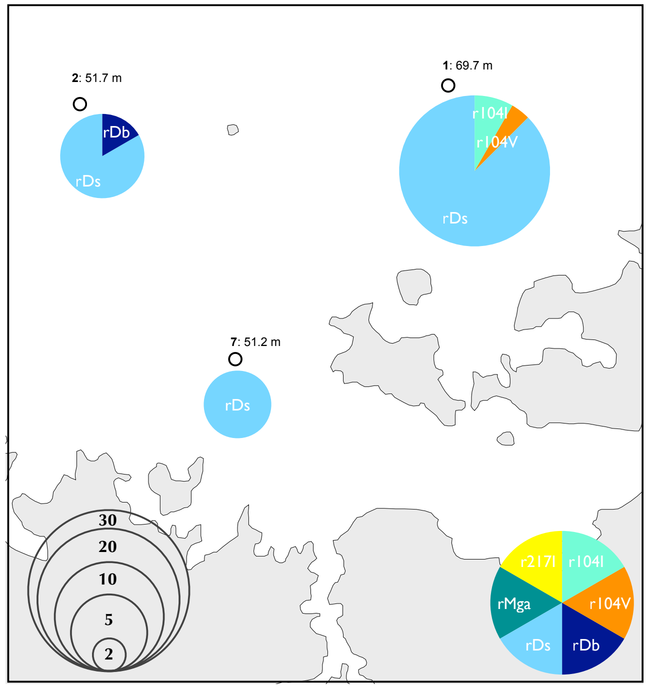

Fig. S1 continued

Figure S1

Short descriptions of the ten species and the frequencies of *LWS* and *RH1* alleles in the populations of (a) *Haplochromis xenognathus*, (b) *H*. sp. 'green dentex', (c) *H*. sp. 'paropius like', (d) *Platytaeniodus degeni*, (e) *H*. sp. 'stone', (f) *H. piceatus*, (g) *H*. sp. cf. hiatus, (h) *H*. sp. 'supramacrops', (i) *H*. sp. 'deepwater cinctus', and (j) *H*. sp. cf. fusiformis are shown in separate panels. Arabic numerals correspond to those in Fig. 1A, and the depths at each point are described on the right side of the numbers. The size of a pie indicates the number of haplotypes sequenced. The standard sizes of pies are shown at the left bottom. The colored sections of a pie indicate the frequency of the correspondent allele in the standard allele color pie (right bottom). The amino acid differences among allele groups are shown in Fig. 2B (LWS) and 2C (RH1). The maps were drawn by Y. T. based on original source maps: https://www.google.com/maps. Fish photographs were taken by M. I. and S. M.
