# Supplementary material for: Visual adaptation in Lake Victoria cichlid fishes: depth-related variation of color and scotopic opsins in species from sand/mud bottoms
Source: BMC Evol Biol. 2017 Aug 22;17:200. doi: 10.1186/s12862-017-1040-x (PMC5568302; doi:10.1186/s12862-017-1040-x)
Supplement: Supplementary file 2 — Figure S2. Alignment of all polymorphic sites of LWS from the ten species. The nucleotide sites are shown on the top of the alignment. “n” and “s” indicate nonsynonymous and synonymous sites, respectively. Dots indicate the nucleotides that are identical with those in the top line. The allele groups of each sequence are shown on the right side of the sequences. (PDF 64 kb) [file 12862_2017_1040_MOESM2_ESM.pdf]

```
             1111112333344455555566666667888888899
             3555684238912601223344677801224589934
             2127649636107534392557469834344531839      allele group
syn./non.    snnnsnsnsnnnnsnnnnnnsnnnnnnnnnnsnsnsn
H allele     CAAGTGCGATTCATCTGGGGCACATGTGTGACGCGTA

12740        .........................A..AT.......      M3/M3
14002        .........................A..AT.......      M3/M3
14794        .........................A..AT.......      M3/M3
14796        .........................A..AT.......      M3/M3
14798        .........................A..AT.......      M3/M3
15876        .........................A..AT.......      M3/M3
15877        .........................A..AT.......      M3/M3
15878        .........................A..AT.......      M3/M3
15879        .........................A..AT.......      M3/M3
15880        .........................A..AT.......      M3/M3
15881        .........................A..AT.......      M3/M3
```

*Haplochromis xenognathus*

```
16798        .........................A..AT.......      M3/M3
16799        .............Y...........R..WK.......      M3/H
16800        .........................R..WK.......      M3/H
16801        .........................A..AT.......      M3/M3
16802        .........................A..AT.......      M3/M3
16803        .........................A..AT.......      M3/M3
16804        ....................SW...A..AT.......      M3/P
16805        .........................A..AT.......      M3/M3
16806        .............Y...........R..WK.......      M3/H
16807        .........................A..AT.......      M3/M3
```

*Haplochromis cf.* 'green dentex'

```
13193        .....AT.G...........GT...A..AT.......      Sp/Sp
13194        .....AT.G...........GT...A..AT.......      Sp/Sp
14495        ........R.KS..S..K.KSWMWWW..AT.......       P/other
14496        .....AT.G...........GT...A..AT.......      Sp/Sp
14545        .....AT.G...........GT...A..AT.......      Sp/Sp
14550        .....AT.G...........GT...A..AT.......      Sp/Sp
14554        .....AT.G...........GT...A..AT.......      Sp/Sp
15607        Y....AT.G.KS..S..K.KGTMWWW..AT.Y.....      Sp/other
15608        .....RY.R...........GT...AY.AT.......      Sp/P
15609        .....RY.R..S........GT...A.RAT.......      Sp/other
15854        .....AT.G...........GT...A..AT.......      Sp/Sp
15865        .....AT.G...........GT...A..AT.......      Sp/Sp
15869        .....AT.G...........GT...A..AT.......      Sp/Sp
15882        .....AT.G...........GT...A..AT.......      Sp/Sp
15898        .....AT.G...........GT...A..AT.......      Sp/Sp
16076        .....AT.G.KS..S..T.T..ATAK..WK.Y.Y..R    2BB/other
16077        .....AT.G...........GT...A..AT.......      Sp/Sp
16138        .....AT.G...........GT...A..AT.......      Sp/Sp
16139        .....RY.G.KS..S..K.KSWMWWW..AT.Y.....      Sp/other
16140        Y....AT.G.KS..S..K.KSWMWWW..AT.Y.....      Sp/2BB
16394        .....RY.............GT...A..AT.......      Sp/P
16395        .....AT.G...........GT...A..AT.......      Sp/Sp
```

*Platytaeniodus degeni*

```
13318        ....................GT...A..AT.......       P/P
13322        ....................GT...A..AT.......       P/P
13323        ....................GT...A..AT.......       P/P
13326        ....................GT...A..AT.......       P/P
13327        ....................GT...A..AT.......       P/P
13331        ....................GT...A..AT.......       P/P
13333        ....................GT...A..AT.......       P/P
```

*Haplochromis* sp. 'stone'

```
11402        ....G...............GT...A..AT.......       P/P
11403        ....G...............GT...A..AT.......       P/P
11407        ....................GT...A..AT.......       P/P
11411        ....................GT...A..AT.......       P/P
11412        ....................GT...A..AT.......       P/P
11495        .T..................GT...A..AT.......       P/P
11496        ....................GT...A..AT.......       P/P
```

*Haplochromis piceatus*

Fig. S2

```
             1111112333344455555566666678888888899
             3555684238912601223344677801224589934
             2127649636107534392557469834344531839      allele group
syn./non.    snnnsnsnsnnnnsnnnnnnsnnnnnnnnnnnsnsnsn
H allele     CAAGTGCGATTCATCTGGGGCACATGTGTGACGCGTA

11500        ....................GT...A..AT.......      P/P
11509        ....................GT...A..AT.......      P/P
11510        ....................GT...A..AT.......      P/P
11515        ....................GT...A..AT.......      P/P
11516        ....................GT...A..AT.......      P/P
11518        ....................GT...A..AT.......      P/P
11520        ....................GT...A..AT.......      P/P
11560        ....................GT...A..AT.......      P/P
11565        ....................GT...A..AT.......      P/P
11568        ....................GT...A..AT.......      P/P

11351        ..............T..........A..ATG......      D/D
11352        ..............Y..........A..ATG......      D/D
11353        ..............T..........A..ATG......      D/D
11374        ..............T..........A..ATG......      D/D
11375        ..............Y...R......A..ATG......      D/D
11377        ..............Y.....SW...A..ATR......      D/D
11378        ................R........A..ATG......      D/D
11381        ..............Y..........A..ATG...R..      D/D
11383        ..............Y.R........A..ATG......      D/D
11386        ................R........A..ATG......      D/D
11534        ...........S..T..........A..ATG......      D/D
11535        .........................A..ATG......      D/D
11536        ....G.........T..........A..ATG......      D/D
14023        ..............Y...R......A..ATG......      D/D
14024        ..............T..........A..ATG......      D/D
14025        ..............Y.R........A..ATG......      D/D

00680        .........................A..ATG...A..      D/D
00681        .........................A..ATG.S.R..      D/D
00697        .........................A..ATG......      D/D
00699        .........................A..ATG...R..      D/D
00700        ..................A......A..ATG......      D/D
00701        ..................A......A..ATG......      D/D
0702         ..................A......A..ATG......      D/D
0703         ..................R......A..ATG......      D/D
0704         ..................A......A..ATG......      D/D
0705         ..................A......A..ATG...R..      D/D
0707         .........................A..ATG...R..      D/D
0708         ..................R......A..ATG......      D/D
0711         ..................A......A..ATG......      D/D
00732        ..................R......A..ATG......      D/D
00733        ...K..............R.........WKR......      D/other
00736        ..................A......A..ATG......      D/D
00737        ................R.R......A..ATG......      D/D
0738         ..................R......A..ATG......      D/D
0740         ..................A......A..ATG......      D/D
0741         ..................A......A..ATG......      D/D
0751         ..................A......A..ATG......      D/D
0752         ..................A......A..ATG......      D/D
1103         ..................R......A..ATG...R..      D/D
1105         ..................R......A..ATG...R..      D/D
1106         ..................A......A..ATG......      D/D
1140         .........................A..ATG......      D/D
01146        ..................R......A..ATG...R..      D/D
01147        ..R...............A......A..ATG......      D/D
01148        ..................A......A..ATG......      D/D
01149        ..................R......A..ATG...R..      D/D
11357        ..................A......A..ATG......      D/D
11365        ..................R......A..ATG...R..      D/D
11390        ..................A......A..ATG......      D/D
13639        ....G.............A......A..ATG......      D/D
```

*Haplochromis piceatus*

*Haplochromis* sp. cf. hiatus

*Haplochromis* sp. cf. 'supramacrops'

Fig. S2 continued

```
             1111112333344455555566666678888888899
             3555684238912601223344677801224589934
             2127649636107534392557469834344531839      allele group
syn./non.    snnnsnsnsnnnnsnnnnnnsnnnnnnnnnnsnsnsn
H allele     CAAGTGCGATTCATCTGGGGCACATGTGTGACGCGTA

00709        ................R........A..ATG......          D/D
00710        ................A........A..ATG......          D/D
00712        ....................SW...A..ATR...R..          D/P
01078        ................R........A..ATG......          D/D
01079        ................A........A..ATG......          D/D
01080        ................R........A..ATG...R..          D/D
01081        .........................A..ATG...R..          D/D
01082        .........................A..ATG.S.R..          D/D
01083        .........................A..ATG......          D/D
01086        ................R........A..ATG...R..          D/D
01130        ....................SW...A..ATR......          D/P
01136        ....................SW...A..ATR......          D/P
01137        .........................A..ATG...R..          D/D
01138        ................R........A..ATG...R..          D/D
01139        ................R........A..ATG...R..          D/D
01141        .........................A..ATG......          D/D
01142        ...............KR........A..ATG......          D/D
01143        ...............WA........A..ATG......          D/D
01145        ................R...SW...A..ATR......          D/P
01297        .........Y......A........A..ATG......          D/D
01302        .W..............R........A..ATR...R..          D/M3
01303        ....................SW...A..ATR......          D/P
01304        .........................A..ATG......          D/D
01305        ................A........A..ATG......          D/D
01306        ................R........A..ATG...R..          D/D
01308        ................R........A..ATG......          D/D
01309        .........................A..ATG......          D/D
01310        ................R........A..ATG...R..          D/D
01485        .........................A..ATG...R..          D/D
01487        .........................A..ATG...R..          D/D
01488        .........................A..ATG...A..          D/D
01489        ................R........A..ATG...R..          D/D
01490        .........................A..ATG...R..          D/D

01076        ..............................G......         Ds/Ds
01077        ..............................G......         Ds/Ds
01282        ..............................G......         Ds/Ds
01283        ..............................G......         Ds/Ds
01285        ...........S........SW.....RWKR......         Ds/other
01286        ..............................G......         Ds/Ds
1287         ..............................G......         Ds/Ds
1289         ..............................G......         Ds/Ds
1290         ..............................G......         Ds/Ds
1291         ..............................G......         Ds/Ds
1292         ..............................G......         Ds/Ds
1294         ................R.............G......         Ds/Ds
1295         ..............................G......         Ds/Ds
1296         ..............................G......         Ds/Ds
1298         ..............................G......         Ds/Ds
1299         ..............................G......         Ds/Ds
01300        ..............................G......         Ds/Ds
```

*Haplochromis* sp. ‘deepwater cinctus’

*Haplochromis* sp. cf. fusiformis

Fig. S2 continued

```
              1111112333344455555566666678888888899
              3555684238912601223344677801224589934
              2127649636107534392557469834344531839      allele group
syn./non.     snnnsnsnsnnnnsnnnnnnsnnnnnnnnnnnsnsnsn
H allele      CAAGTGCGATTCATCTGGGGCACATGTGTGACGCGTA

11022         T....AT.G.GG..G..T.T..ATAT..AT.T.....        2BB/2BB
11023         T....AT.G.GG..G..T.T..ATAT..AT.T.....        2BB/2BB
11029         T....AT.G.GG..G..T.T..ATAT..AT.T.....        2BB/2BB
13068         T....AT.G.GG..G..T.T..ATAT..AT.T.....        2BB/2BB
13070         T....AT.G.GG..G..T.T..ATAT..AT.T.....        2BB/2BB
13384         T....AT.G.GG..G..T.T..ATAT..AT.T.....        2BB/2BB
13385         T....AT.G.GG..G..T.T..ATAT..AT.T.....        2BB/2BB
13388         T....AT.G.GG..G..T.T..ATAT..AT.T.....        2BB/2BB
14497         T....AT.G.GG..G..T.T..ATAT..AT.T.....        2BB/2BB
14506         T....AT.G.GG..G..T.T..ATAT..AT.T.....        2BB/2BB
14508         T....AT.G.GG..G..T.T..ATAT..AT.T.....        2BB/2BB
14509         T....AT.G.GG..G..T.T..ATAT..AT.T.....        2BB/2BB
14510         T....AT.G.GG..G..T.T..ATAT..AT.T.....        2BB/2BB
14977         T....AT.G.GG..G..T.T..ATAT..AT.T.....        2BB/2BB
14978         T....AT.G.GG..G..T.T..ATAT..AT.T.....        2BB/2BB
15619         T....AT.G.GG..G..T.T..ATAT..AT.T.....        2BB/2BB
15620         T....AT.G.GG..G..T.T..ATAT..AT.T.....        2BB/2BB
15737         T....AT.G.GG..G..T.T..ATAT..AT.T.....        2BB/2BB
15751         T....AT.G.GG..G..T.T..ATAT..AT.T.....        2BB/2BB
15752         T....AT.G.GG..G..T.T..ATAT..AT.T.....        2BB/2BB
16084         T....AT.G.GG..G..T.TSWATAT.RAT.T.....        2BB/other
16085         T....AT.G.GG..G..T.T..ATAT..AT.T.....        2BB/2BB
16086         T....AT.G.GG..G..T.T..ATAT..AT.T.....        2BB/2BB
16087         T....AT.G.GG..G..T.T..ATAT..AT.T.....        2BB/2BB
16088         T....AT.G.GG..G..T.T..ATAT..AT.T.....        2BB/2BB
16089         T....AT.G.GG..G..T.T..ATAT..AT.T.....        2BB/2BB
16090         T....AT.G.GG..G..T.T..ATAT..AT.T.....        2BB/2BB
16091         T....AT.G.GG..G..T.T..ATAT..AT.T.....        2BB/2BB
16092         T....AT.G.GG..G..T.T..ATAT.RAT.T.....        2BB/2BB
16093         T....AT.G.GG..G..T.T..ATAT..AT.T.....        2BB/2BB
16237         T....AT.G.GG..G..T.T..ATAT..AT.T.....        2BB/2BB
16238         Y....RY....S..S.RK.K..MWWW.RATRY.....          D/other
16239         T....AT.G.GG..G..T.T..ATAT..AT.T.....        2BB/2BB
16240         T....AT.G.GG..G..T.T..ATAT..AT.T.....        2BB/2BB
16241         T....AT.G.GG..G..T.T..ATAT..AT.T.....        2BB/2BB
16242         T....ATSG.GG..G..T.T..ATAT..WK.T.....        2BB/2BB
16243         T....AT.G.GG..G..T.T..ATAT..AT.T.....        2BB/2BB
16244         T....AT.G.GG..G..K.K..ATAT.AAT.T.....        2BB/2BB
16245         Y....RY....S..SKRK.K..MWWW..ATRY.....        2BB/D
16246         T....ATSG.GG..G..T.T..ATAT..WK.T.....        2BB/other
17285         Y....RY.R.KS..S.RK.K..MWWW.RATRY...Y.          D/other
17286         Y....RY.G.GS..S.RK.K..MWWW..ATRY.....        2BB/D
17287         ............R...R...SW...A..ATR......          D/P
17288         T....AT.G.GG..G..T.T..ATAT..AT.T.....        2BB/2BB
```

*Haplochromis* sp. 'paropius like'

**Figure S2**

Alignment of all polymorphic sites of *LWS* from the ten species. The nucleotide sites are shown on the top of the alignment. "n" and "s" indicate nonsynonymous and synonymous sites, respectively. Dots indicate the nucleotides that are identical with those in the top line. The allele groups of each sequence are shown on the right side of the sequences.

Fig. S2 continued
