# Supplementary material for: Visual adaptation in Lake Victoria cichlid fishes: depth-related variation of color and scotopic opsins in species from sand/mud bottoms
Source: BMC Evol Biol. 2017 Aug 22;17:200. doi: 10.1186/s12862-017-1040-x (PMC5568302; doi:10.1186/s12862-017-1040-x)
Supplement: Supplementary file 3 — Figure S3. An alignment of all polymorphic sites of RH1 from 10 species used in this study. The nucleotide sites are shown on top of the alignment. “n” and “s” indicate nonsynonymous and synonymous sites, respectively. Dots indicate where nucleotides are identical with those in the top line. The allele groups of each sequence are shown right side of the sequences. (PDF 62 kb) [file 12862_2017_1040_MOESM3_ESM.pdf]

```
                 1112233344444455666666788889
              6792274815977888811333558805691
              9084533307735148979789027756822      allele group
syn./non.     snnnnnsnnsnnnnnnsnsnnsnnsnnnnns
r104V         TTGTGCGGGTGGGTAGGCTTTACATAGGCGT
```

Haplochromis xenognathus

```
12740         ........A......................       r104I/r104I
14003         ........A......................       r104I/r104I
14005         ........A......................       r104I/r104I
14794         ........A......................       r104I/r104I
14796         ........A......................       r104I/r104I
14798         ........A......................       r104I/r104I
15876         ........A......................       r104I/r104I
15877         ........A.......K..............       r104I/r104I
15878         ........R......................       r104I/r104V
15879         ........A......................       r104I/r104I
15880         ........A......................       r104I/r104I
15881         ........A......................       r104I/r104I
```

Haplochromis sp. cf. 'green dentex'

```
16798         ........A......................       r104I/r104I
16799         --......A......................       r104I/r104I
16800         ........A......................       r104I/r104I
16801         ........A......................       r104I/r104I
16802         ........A......................       r104I/r104I
16803         ........A......................       r104I/r104I
16804         ...............................       r104V/r104V
16805         ........A......................       r104I/r104I
16806         ......R.A......................       r104I/r104I
16807         ........A......................       r104I/r104I
```

Platytaeniodus degeni

```
13193         ..R.....A......................       r104I/r104I
13194         ........A......................       r104I/r104I
14495         ..R.....A......................       r104I/r104I
14496         ........A......................       r104I/r104I
14545         ........A......................       r104I/r104I
14550         ........A......................       r104I/r104I
14554         ........A......................       r104I/r104I
15607         ........A......................       r104I/r104I
15608         ........A......................       r104I/r104I
15609         ........A......................       r104I/r104I
15854         ........A......................       r104I/r104I
15865         ........A......................       r104I/r104I
15869         ........A......................       r104I/r104I
15882         ........A......................       r104I/r104I
15898         ........A......................       r104I/r104I
16076         ..R.....A......................       r104I/r104I
16077         ........A......................       r104I/r104I
16138         ..R.....A......................       r104I/r104I
16139         ........A......................       r104I/r104I
16140         ..R.....A......................       r104I/r104I
16394         ........A......................       r104I/r104I
16395         ........A......................       r104I/r104I
```

Haplochromis sp. 'stone'

```
13318         ........A......................       r104I/r104I
13322         ........A......................       r104I/r104I
13323         ........A......................       r104I/r104I
13327         ........A......................       r104I/r104I
13331         ........A......................       r104I/r104I
13333         ........A......................       r104I/r104I
```

Haplochromis piceatus

```
11402         ...........T..............A....         rDs/rDs
11403         .........................GA....         rDs/rDs
11407         ...........T..............A....         rDs/rDs
11411         ..........................R....         rDs/r104V
11412         ........R..K..............A....         rDs/rDs
11495         ...........T..............A....         rDs/rDs
11496         ...GC....Y................A....         rDs/rDs
11500         ...........T..............A....         rDs/rDs
11501         ...........T..............A....         rDs/rDs
```

Fig. S3

```
                1112233344444455666666788889
             6792274815977888811333558805691
             9084533307735148979789027756822      allele group
syn./non.    snnnnnsnnsnnnnnnsnsnnsnnsnnnnns
r104V        TTGTGCGGGTGGGTAGGCTTTACATAGGCGT

11509        ...........T..............A....        rDs/rDs
11510        .........Y.K..............A....        rDs/rDs
11515        ...........K..............A....        rDs/rDs
11516        ...........T..............A....        rDs/rDs
11518        .........Y.K..............A....        rDs/rDs
11520        ...........K..............A....        rDs/rDs
11560        ........R..K..............A.M..        rDs/rDs
11565        ...........T..............A....        rDs/rDs
11568        ...KS....Y.K..............A....        rDs/rDs
```

Haplochromis piceatus

```
11351        ...............................      r104V/r104V
11352        ...............................      r104V/r104V
11353        ...............................      r104V/r104V
11374        ...............................      r104V/r104V
11375        ...............................      r104V/r104V
11377        ...............................      r104V/r104V
11378        ...............................      r104V/r104V
11381        WK...S.........................      r104V/r104V
11383        ...............................      r104V/r104V
11386        ...............................      r104V/r104V
11534        ...............................      r104V/r104V
11535        ...............................      r104V/r104V
11536        ...............................      r104V/r104V
14023        ...............................      r104V/r104V
14024        ...............................      r104V/r104V
14025        ...............................      r104V/r104V
```

Haplochromis sp. cf. hiatus

```
00680        .............................T.       rMga/rMga
00681        .............................T.       rMga/rMga
00682        .............................T.       rMga/rMga
00697        .............................T.       rMga/rMga
00699        .............................T.       rMga/rMga
00700        ........................Y....K.       rMga/r104V
00701        .............................T.       rMga/rMga
00703        .............................T.       rMga/rMga
00704        ........R....................K.       rMga/r104V
00705        .............................T.       rMga/rMga
00707        .............................T.       rMga/rMga
00708        .............................T.       rMga/rMga
00711        .............................T.       rMga/rMga
00732        .............................T.       rMga/rMga
00733        .............................T.       rMga/rMga
00736        .............................T.       rMga/rMga
00737        .............................T.       rMga/rMga
00738        .............................K.       rMga/r104V
00739        .............................T.       rMga/rMga
00740        ...............................      r104V/r104V
00741        .............................K.       rMga/r104V
00751        .............................T.       rMga/rMga
00752        .............................T.       rMga/rMga
01103        ........R....................K.       rMga/r104V
01105        ........................Y....K.       rMga/r104V
01106        .............................T.       rMga/rMga
01140        ........................Y....K.       rMga/r104V
01146        .............................K.       rMga/r104V
01147        .............................T.       rMga/rMga
01148        .............................T.       rMga/rMga
01149        .............................T.       rMga/rMga
11357        .............................T.       rMga/rMga
11363        .............................T.       rMga/rMga
11365        .............................T.       rMga/rMga
11390        .............A..........Y....T.       rMga/rMga
13639        .............................T.       rMga/rMga
```

Haplochromis sp. cf. ‘supramacrops’

Fig. S3 continued

```
                 1112233344444455666666788889
                679227481597788881133355880569 1
                9084533307735148979789027756822      allele group
syn./non.       snnnnnsnnsnnnnnnsnsnnsnnsnnnnns
r104V           TTGTGCGGGTGGGTAGGCTTTACATAGGCGT

00709           ...GC..A.Y...............GA....         rDb/rDb
00710           ...KS..R.................RR....         rDb/r104V
00712           ...GC..A..R..............GA...Y         rDb/rDb
00716           ...GC..A.................GA...Y         rDb/rDb
01078           ...GC..A..A..............GA...C         rDb/rDb
01079           ...GC..A..R..............GA...C         rDb/rDb
01080           ...GC..A..A..............GA...C         rDb/rDb
01081           ...GC..A..A..............GA...C         rDb/rDb
01082           ...GC..A.YR..............GA...C         rDb/rDb
01083           ...GC..A..R..............GA...Y         rDb/rDb
01086           ...GC..A.YR..............GA...C         rDb/rDb
01130           ...GC..A.Y...............GA....         rDb/rDb
01136           ...GC..A..A..............GA...C         rDb/rDb
01137           ...GC..A.C...............GA....         rDb/rDb
01138           ...GC..A..R..............GA...C         rDb/rDb
01139           ...GC..A..A..............GA...C         rDb/rDb
01141           ...GC..A.Y...............GA...Y         rDb/rDb
01142           ...GC..A..R..............GA...C         rDb/rDb
01143           ...GC..A..R..............GA...Y         rDb/rDb
01145           ...GC..A..A..............GA...C         rDb/rDb
01297           ...GC..A..A..............GA...C         rDb/rDb
01304           ...GC..A..A..............GA...C         rDb/rDb
01305           ...GC..A..A..............GA...C         rDb/rDb
01306           ...GC..A..A..............GA...C         rDb/rDb
01308           ...GC..A.Y...............GA...C         rDb/rDb
01309           ...GC..A..R..............GA...C         rDb/rDb
01310           ...GC..A..A..............GA...C         rDb/rDb
01485           ...GC..A..A..............GA...C         rDb/rDb
01487           ...GC..A..A..............GA...C         rDb/rDb
01488           ...GC..A.................GA....         rDb/rDb
01489           ...GC..A..A..............GA...C         rDb/rDb
01490           ...GC..A.YR..............GA...C         rDb/rDb

01076           ...........T..............A....         rDs/rDs
01077           ...........T..............A....         rDs/rDs
01282           ...........T..............A....         rDs/rDs
01283           ...........T..............A....         rDs/rDs
01285           ...........T..............A....         rDs/rDs
01286           ...........T..............A....         rDs/rDs
01287           ........R.................R....         rDs/r104I
01289           ...........T..............A....         rDs/rDs
01290           ...........T..............A....         rDs/rDs
01291           ...........K..............R....         rDs/r104V
01292           ...........T..............A....         rDs/rDs
01294           ...KS......K..............A....         rDs/rDs
01295           ...........T..............A....         rDs/rDs
01296           ........R..K..............R....         rDs/r104I
01298           ...........T..............A....         rDs/rDs
01299           ...KS....Y.YK.RC.SYWYR.R.RA...C         rDs/rDb
01300           ...........T..............A....         rDs/rDs
```

Haplochromis sp. ‘cinctus’

Haplochromis sp. cf. fusiformis

Fig. S3 continued

```
                 1112233344444455666666788889
              6792274815977888811333558805691
              9084533307735148979789027756822      allele group
syn./non.     snnnnnsnnsnnnnnnsnsnnsnnsnnnnns
r104V         TTGTGCGGGTGGGTAGGCTTTACATAGGCGT

11022         ......................Y........       r217I/r104V
11023         ......................T........       r217I/r217I
11029         ......................Y........       r217I/r104V
13068         ......................T........       r217I/r217I
13070         ......................Y........       r217I/r104V
13384         ......................T........       r217I/r217I
13385         ......................Y........       r217I/r104V
13386         ................K.....T........       r217I/r217I
13387         ......................T........       r217I/r217I
14497         ......................T........       r217I/r217I
14506         ..........R...........Y........       r217I/r104V
14508         ......................T........       r217I/r217I
14509         ......................T........       r217I/r217I
14510         ......................T........       r217I/r217I
14977         ......................T........       r217I/r217I
15619         ......................Y........       r217I/r104V
15620         ......................Y........       r217I/r104V
15734         ......................Y........       r217I/r104V
15737         ......................T........       r217I/r217I
15751         ......................Y........       r217I/r104V
15752         ......................Y........       r217I/r104V
16084         ......................Y........       r217I/r104V
16085         ......................Y........       r217I/r104V
16086         ......................Y........       r217I/r104V
16087         ......................T........       r217I/r217I
16088         ......................Y........       r217I/r104V
16089         ......................T........       r217I/r217I
16090         ......................T........       r217I/r217I
16091         ......................T........       r217I/r217I
16092         ......................T........       r217I/r217I
16093         ................K.....T........       r217I/r217I
16237         ..........R.....K.....T........       r217I/r217I
16238         ......................T........       r217I/r217I
16239         ......................T........       r217I/r217I
16240         ......................T........       r217I/r217I
16241         ......................Y....K...       r217I/r104V
16242         ......................Y........       r217I/r104V
16243         ......................T........       r217I/r217I
16244         ......................T........       r217I/r217I
16245         ......................Y........       r217I/r104V
16246         ................K.....T........       r217I/r217I
17285         ......................Y........       r217I/r104V
17286         ......................T........       r217I/r217I
17287         ................K.....T........       r217I/r217I
17288         ......................T........       r217I/r217I
```

Haplochromis sp. 'paropius like'

Figure S3

An alignment of all polymorphic sites of *RH1* from 10 species used in this study. The nucleotide sites are shown on top of the alignment. "n" and "s" indicate nonsynonymous and synonymous sites, respectively. Dots indicate where nucleotides are identical with those in the top line. The allele groups of each sequence are shown right side of the sequences.

Fig. S3 continued
