# Supplementary material for: Visual adaptation in Lake Victoria cichlid fishes: depth-related variation of color and scotopic opsins in species from sand/mud bottoms
Source: BMC Evol Biol. 2017 Aug 22;17:200. doi: 10.1186/s12862-017-1040-x (PMC5568302; doi:10.1186/s12862-017-1040-x)
Supplement: Supplementary file 4 — Table S1. A2 ratio in RH1 and LWS pigments estimated from MSP data. (PDF 119 kb) [file 12862_2017_1040_MOESM4_ESM.pdf]

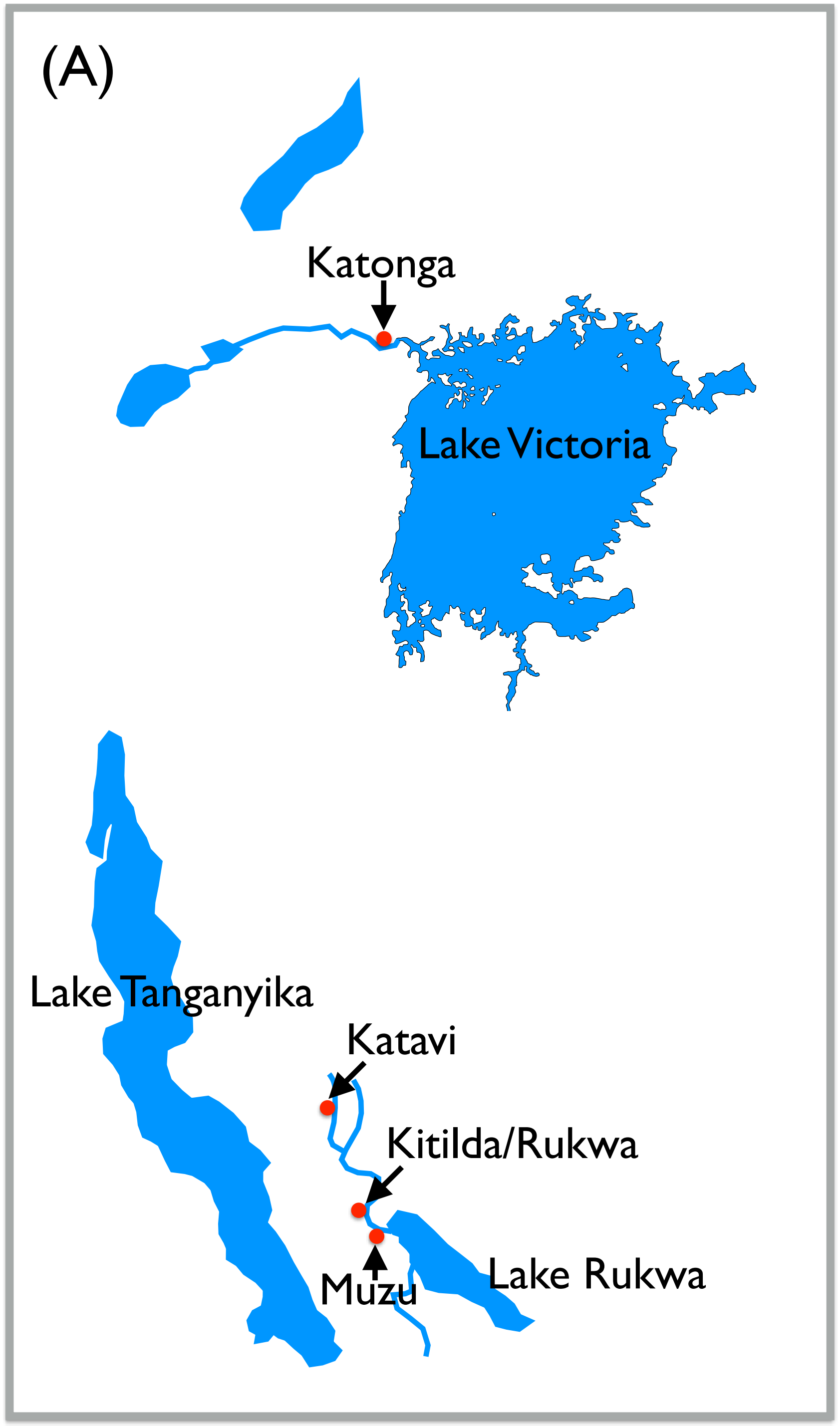

(B)

Amino acid positions	62	168	177	179	191	222	226	227	317	
H	V	A	A	V	Y	L	I	F	I	
8676	.	.	.	.	.	.	.	.	.	Katonga
8684_A1	.	.	.	.	.	.	.	.	.	
8684_A2	I	G	S	F	F	I	F	I	V	
9317	I	G	S	F	F	I	F	I	V	Katavi
9334_A1	I	.	.	.	.	I	F	I	V	Kitilda/Rukwa
9334_A2	I	G	S	F	F	I	F	I	V	
9354	I	G	S	F	F	I	F	I	V	
9355	.	.	.	.	.	.	.	.	.	Muzu
9379	I	G	S	F	F	I	F	I	V	
9380	I	G	S	F	F	I	F	I	V	

Figure S4

Amino acid alignment of LWS from river species. Residue positions are numbered according to the sequences of LWS. The dots and letters indicate identical and different residues, respectively, compared with the top line. The *LWS* sequences were determined from four riverine species: *H*. sp. ‘katonga’ (n = 2), *H*. sp. ‘katavi’ (n = 1), *H*. sp. ‘kitilda-rukwa’ (n = 2), and *H*. sp. ‘muzu-rukwa’ (n = 3).
