# Supplementary material for: Visual adaptation in Lake Victoria cichlid fishes: depth-related variation of color and scotopic opsins in species from sand/mud bottoms
Source: BMC Evol Biol. 2017 Aug 22;17:200. doi: 10.1186/s12862-017-1040-x (PMC5568302; doi:10.1186/s12862-017-1040-x)
Supplement: Supplementary file 5 — Figure S4. Amino acid alignment of LWS from river species. Residue positions are numbered according to the sequences of LWS. The dots and letters indicate identical and different residues, respectively, compared with the top line. The LWS sequences were determined from four riverine species: H. sp. ‘katonga’ (n = 2), H. sp. ‘katavi’ (n = 1), H. sp. ‘kitilda-rukwa’ (n = 2), and H. sp. ‘muzu-rukwa’ (n = 3). (PDF 22 kb) [file 12862_2017_1040_MOESM5_ESM.pdf]

Table S1. A2 ratio in RH1 and LWS pigments estimated from MSP data

Species	RH1 allele	MSP	A2 ratio (%)
Pundamilia nyererei (Python)[1]	r104V	505 nm	20
Haplochromis pyrrhocephalus[2]	r104V*	519 nm	84

Species	LWS allele	MSP	A2 ratio (%)
Pundamilia nyererei (Python)[1]	H	567 nm	30
P. nyererei (Python)[1]	H	571 nm	41
Haplochromis piceatus[2]	P*	565 nm	57
H. pyrrhocephalus[2]	Py*	595 nm	100

1: Carleton et al. 2005, 2: van der Meer, Bowmaker 1995. * Allele was estimated from this study.
