# Supplementary material for: Visual adaptation in Lake Victoria cichlid fishes: depth-related variation of color and scotopic opsins in species from sand/mud bottoms
Source: BMC Evol Biol. 2017 Aug 22;17:200. doi: 10.1186/s12862-017-1040-x (PMC5568302; doi:10.1186/s12862-017-1040-x)
Supplement: Supplementary file 6 — Diagnosis and remarks of species used in this study. (DOCX 21 kb) [file 12862_2017_1040_MOESM6_ESM.docx]

Additional text. Diagnosis and remarks of species used in this study

***Haplochromis* sp. cf. ‘green dentex’**

*Haplochromis* sp. cf. ‘green dentex’ recently may become much smaller than the past, because standard length of the species was smaller than 90 mm except for one largest individual, 120 mm, whereas specimens in HEST collection are much larger, 120-145 mm standard length (van Oijen, personal communication). Piscivorous haplochromine are morphologically identified by the combination of acutely pointed unicuspid teeth in outer rows of the oral jaws (vs. bluntly pointed and unequally bicuspid) and lower jaw length more than 45% of head length (Witte and van Oijen, 1995). In our survey, lower jaw length of the recently collected specimens were not so large, 40.5-45.0% of head length except for one largest specimen (48.3%), it may be positive allometry. It is necessary to compare the same sized specimens at pre- and post-Nile perch upsurge.

***Haplochromis* sp. ‘paropius like’**

*Haplochromis* sp. ‘paropius like’ is one of insectivores/detritivores species complex (Witte and van Oijen, 1995), which is distinguished from the other remnant haplochromines by the combination of smaller body which is generally less than 80 mm standard length, straight or moderately curved dorsal head profile (vs. curved or concave), unequally bicuspid outer teeth in oral jaws (vs. unicuspid or weakly bicuspid), 2-3 inner teeth rows which are separating from outer teeth row (vs. more than three rows and closely set), and general-sized scales on chest (vs. smaller). This species complex includes a lot of species and quite difficult to identify, because there is few morphological differences except for coloration of mature male. Formerly most of the species complex was described as *Enterochromis* Greenwood 1986, by highly coiled long enteric canal (more than twice of standard length), however recently they do not have such a long enteric canal, it would be adaptation for dietary change (Kishe-Machumu et al., 2008 and de Zeeuw et al., 2010). Among the species complex, this species is distinguished by the combination of orange coloration and broad mid-lateral band on lateral body in mature male.

***Haplochromis* sp. ‘stone’**

*Haplochromis* sp. ‘stone’ is distinguished from other haplochromines which have molariform teeth on the lower pharyngeal bone (so called “pharyngeal mollusc crushers” in Witte and van Oijen, 1995) by the combination of deep body (vs. slender to moderately deep), stout, rounded, unicuspid and lesser number of outer teeth in oral jaw (vs. slender, moderately flattened, unequally bicuspid and more numbers), and red large distinct spots on caudal fin in mature male (vs. absent, indistinct or small). In this study only specimens from offshore islands were examined while the species are distributed also in coastal zone, because specimen numbers in the same locality were not enough for this study. The specimens from coastal zone look somewhat different from that from offshore islands (used in this study), but we consider them as the same species, because no morphological difference was observed at the moment. Further taxonomical study is necessary for the species.

***Haplochromis* *piceatus***

*Haplochromis* *piceatus* is distinguished from insectivores/detritivores species complex by more number of gill rakers on lower limb of gill arch (generally the number is 11 or 12, whereas less than 11 in insectivores/detritivores, except for one undescribed species, which was not used in this study) and tricuspid outer teeth existing together with bicuspid teeth in posterior margin of upper jaw (vs. only bicuspid), and black to dark-green body coloration with yellow to orange unpaired fins. Also this species is distinguished from zooplanktivorous by the combination of slightly prominent premaxillary pedicel (vs. not or weakly prominent), relatively deeper body, and presence of outer teeth in posterior end of premaxilla (generally absent), and its coloration in mature male. Coloration of the species was not described in original description, but Dr. Witte gave information.

***Haplochromis* sp. cf. hiatus**

*Haplochromis* sp. cf. hiatus is distinguished from remnant insectivores/detritivores by the combination of relatively deeper body (vs. moderately deep to slender), weakly bicuspid and/or unequally bicuspid outer teeth in oral jaw (vs. unequally bicuspid only), slightly prominent premaxillary pedicel (vs. not or weakly prominent), and black or dark green coloration in mature male. However these characters are quite unclear in smaller individual and impossible to distinguish from the other detritivores/insectivores.

***Haplochromis* sp. cf. ‘supramacrops’**

*Haplochromis* sp. cf. ‘supramacrops’ is identified morphologically as Zooplanktivorous and distinguished from the other remnant zooplanktivores by the combination of large eye whose rim is reaching or over the dorsal head profile in lateral view (vs. not reaching), and dark-green body and dark-red caudal fin in mature male. Three described haplochromines have such a large eye (*H*. *cinereus*, *H*. *macrops* and *H*. *megalops*) but *Haplochromis* sp. cf. ‘supramacrops’ is distinguished from these three species by combination of slender body, narrow interorbital width, slightly smaller mouth, more number of gill rakers on lower limb of gill arch, and unequally bicuspid outer teeth. The former two species have never been reported from southern side of the lake, and the latter is recently very rare (IUCN red list, 2013).

***Haplochromis* sp. ‘deepwater cinctus’**

*Haplochromis* sp. ‘deepwater cinctus’ is distinguished from remnant insectivores/detritivores by the combination of small and relatively slender body, weakly bicuspid and/or unequally bicuspid outer teeth in oral jaws (vs. unequally bicuspid only), and red body coloration in mature male. This species is morphologically intermediate between two remnant species, *H.* sp. cf. iris and *H.* sp. cf. cinctus (both of the species were possibly the same to *H. iris* and *H. cinctus* respectively, but we cannot predicate identification at the moment because recently collected specimens showed slight morphological difference comparing to original description). These three species have similar red coloration, but *H* sp. ‘deepwater cinctus’ is different from *H.* sp. cf. iris by slightly slender body and different from *H.* sp. cf. cinctus by slightly larger head and its dentition (only unequally bicuspid teeth present in *H*. sp. cf. cinctus), however the characters is overlapped and difficult to distinguish in individual level. Further taxonomy is necessary for the species complex.

***Haplochromis* sp. cf. fusiformis**

*Haplochromis* sp. cf. fusiformis is morphologically identified as Zooplanktivores by slender body and bicuspid teeth in oral jaws. No other described species has such a slender body (body depth less than 26% standard length) except for *H. fusiformis.* These two species are different only in its coloration; *H.* sp. cf. fusiformis has pale dark-green coloration in mature male, whereas *H. fusiformis* is ‘brilliant purple-blue, shading to silvery-yellow on the flank’ according to original description. They are possibly local variation of single species or sister species. Recently *H. fusiformis* is abundant in some locality at Uganda side, northwestern part of the lake (Namulemo, 1999) but no report from Tanzanian side, southern part of the lake.

References

Namulemo, G. (1999) Species composition and relative abundance of zooplanktivorous haplochromines in the northern portion of Lake Victoria (Uganda). In: Report on Fourth FIDAWOG Workshop held at Kisumu, 16 to 20 August 1999. Jinja, Uganda, Lake Victoria Fisheries Research Project, pp. 199-203. (LVFRP Technical Document,7)

Witte, F., de Zeeuw, M.P. & Brooks, E. 2010. *Platytaeniodus* *degeni*. *In*: IUCN 2013. IUCN Red List of Threatened Species. Version 2013.2. <www.iucnredlist.org>. Downloaded on 27 December 2013.

IUCN 2013. The IUCN Red List of Threatened Species. Version 2013.2. <http://www.iucnredlist.org>. Downloaded on 21 November 2013.

Barel, C. D. N., Van Oijen, M. J. P., Witte, F., & Witte-Maas, E. L. (1976). An introduction to the taxonomy and morphology of the haplochromine Cichlidae from Lake Victoria. Netherlands journal of zoology, 27(4), 333-380.

Kishe-Machumu, M., Witte, F., & Wanink, J. H. (2008). Dietary shift in benthivorous cichlids after the ecological changes in Lake Victoria. Animal Biology, 58(4), 401-417.

Seehausen , O. , Witte , F. , Katunzi , E.F.B. , Smits , J. & Bouton , N. ( 1997b ). Patterns of the remnant cichlid

fauna in southern Lake Victoria . Conserv. Biol. , 11 , 890 - 904 .

Witte, F. and van Oijen MJP. Bilogy of haplochromne trophic groups. In: Witte, F., & Densen, W. V. (1995) eds. Fish stocks and fisheries of Lake Victoria. A handbook for field observations.

De Zeeuw, M. P., Mietes, M., Niemantsverdriet, P., Ter Huurne, S., & Witte, F. (2010). Seven new species of detritivorous and phytoplanktivorous haplochromines from Lake Victoria. Zool Med, 84, 201-250.
